# Understanding Sources and Drivers of Size-Resolved Aerosol in the High Arctic Islands of Svalbard Using a Receptor Model Coupled with Machine Learning

**DOI:** 10.1021/acs.est.1c07796

**Published:** 2022-07-25

**Authors:** Congbo Song, Silvia Becagli, David C. S. Beddows, James Brean, Jo Browse, Qili Dai, Manuel Dall’Osto, Valerio Ferracci, Roy M. Harrison, Neil Harris, Weijun Li, Anna E. Jones, Amélie Kirchgäßner, Agung Ghani Kramawijaya, Alexander Kurganskiy, Angelo Lupi, Mauro Mazzola, Mirko Severi, Rita Traversi, Zongbo Shi

**Affiliations:** † School of Geography, Earth and Environment Sciences, University of Birmingham, Birmingham B15 2TT, U.K.; ‡ Department of Chemistry “Ugo Schiff”, University of Florence, Via della Lastruccia 3, Sesto Fiorentino 50019, Italy; § National Research Council of Italy, Institute of Polar Sciences (CNR-ISP), Via Torino 155, Venice-Mestre 30172, Italy; ∥ National Centre for Atmospheric Science (NCAS), School of Geography, Earth and Environmental Sciences, University of Birmingham, Birmingham B15 2TT, U.K.; ⊥ Centre for Geography and Environmental Science, University of Exeter, Penryn TR10 9FE, U.K.; # State Environmental Protection Key Laboratory of Urban Ambient Air Particulate Matter Pollution Prevention and Control, College of Environmental Science and Engineering, Nankai University, Tianjin 300350, China; ¶ Institute of Marine Science, Consejo Superior de Investigaciones Científicas (CSIC), Barcelona 08003, Spain; ∇ Centre for Environmental and Agricultural Informatics, School of Water, Energy & Environment, Cranfield University, College Road, Cranfield MK43 0AL, U.K.; ○ Department of Atmospheric Sciences, School of Earth Sciences, Zhejiang University, Hangzhou 310027, China; ⧫ British Antarctic Survey, Natural Environment Research Council, Cambridge CB3 0ET, U.K.; †† National Research Council of Italy, Institute of Polar Sciences (CNR-ISP), Via P. Gobetti 101, 40129 Bologna, Italy; ‡‡ Department of Environmental Sciences, Faculty of Meteorology, Environment and Arid Land Agriculture, King Abdulaziz University, Jeddah, 21589, Saudi Arabia

**Keywords:** Arctic, source apportionment, positive matrix factorization, machine learning, particle number concentration, meteorology

## Abstract

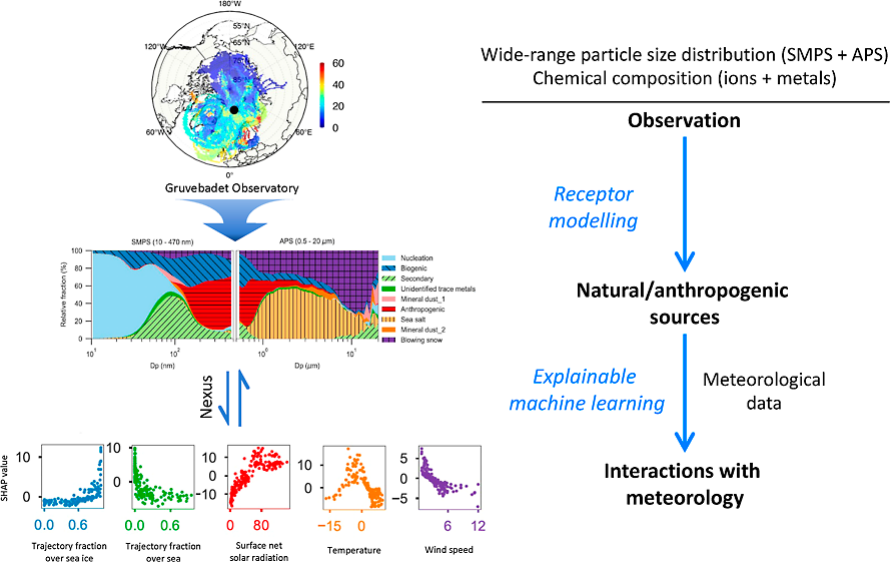

Atmospheric aerosols are important drivers of Arctic climate change through aerosol–cloud–climate interactions. However, large uncertainties remain on the sources and processes controlling particle numbers in both fine and coarse modes. Here, we applied a receptor model and an explainable machine learning technique to understand the sources and drivers of particle numbers from 10 nm to 20 μm in Svalbard. Nucleation, biogenic, secondary, anthropogenic, mineral dust, sea salt and blowing snow aerosols and their major environmental drivers were identified. Our results show that the monthly variations in particles are highly size/source dependent and regulated by meteorology. Secondary and nucleation aerosols are the largest contributors to potential cloud condensation nuclei (CCN, particle number with a diameter larger than 40 nm as a proxy) in the Arctic. Nonlinear responses to temperature were found for biogenic, local dust particles and potential CCN, highlighting the importance of melting sea ice and snow. These results indicate that the aerosol factors will respond to rapid Arctic warming differently and in a nonlinear fashion.

## Abstract 2

Responses of high-latitude particle number concentrations to environmental drivers are nonlinear and source and process dependent.

## 1 Introduction

The Arctic is a unique region undergoing tremendous environmental changes at a much faster pace than the global average.^1^ Arctic aerosols play an important role in radiative forcing, cloud formation and climate change.^2−4^ Particle number and size distributions, in addition to chemical composition, determine their climate forcing properties and are source and process dependent.^5,6^ Early studies on particle number size distributions (PNSD) in the High Arctic focused on the characteristics of nucleation (10–25 nm in diameter), Aitken (25–100 nm in diameter) and accumulation (100 nm–1 μm in diameter)—mode aerosols.^6,7^ A high occurrence frequency of accumulation-mode aerosols from winter to early spring was attributed to anthropogenic Arctic haze and nucleation/Aitken-mode aerosols in summer to new particle formation (NPF).^6−8^ However, the measured aerosols with diameters less than 500 nm provide little useful information on sea salt, blowing snow and mineral dust as they mainly occur in the coarse mode (1–20 μm in diameter). Only recently, a study has investigated particle volume size distributions (PVSD, diameters from 0.5 to 20 μm) in the Arctic and linked them to coarse-mode anthropogenic, marine and mineral dust particles.^5^ However, the contributions from various sources to total particle number concentration (PN) are still not clear, leading to high uncertainties in quantifying their impacts on the regional climate. Understanding PNSD/PVSD from nucleation mode to coarse mode can shed new light on aerosol sources and processes in the Arctic.

*k*-Means cluster analysis is widely used to semi-quantitatively interpret PNSD/PVSD on the basis of occurrence frequency.^5−8^ However, this method could not apportion aerosols to specific sources because each cluster includes multiple sources.^5^ Positive matrix factorization (PMF) can quantitatively separate source-related factors for both number and volume/mass concentrations.^9−12^ While there are a few studies of source apportionment of Arctic aerosols in mass,^9,12^ no studies to date have quantified source contributions to PN, which is crucial for cloud formation. Furthermore, previous studies suggested that annual cycles of Arctic aerosols with different diameters are governed by different processes.^4,13^ The limited knowledge on different processes controlling size-resolved/source-specific aerosols means that climate models are still unable to realistically reproduce processes in the rapidly changing Arctic.

Here, we conducted the first source apportionment study of both particle composition and size distributions (10 nm–20 μm) with PMF at the Gruvebadet Observatory in Svalbard in 2015. Environmental factors driving source-specific aerosol processes were then explored using the SHapley Additive exPlanation (SHAP) approach,^14,15^ which is an explainable machine learning technique (see the section Materials and Methods).

## 2 Materials and Methods

### 2.1 Sampling and Measurements

Aerosol sampling and measurements were performed from March to September 2015 at the Gruvebadet (GVB) Observatory (78.918 °N, 11.895 °E; 61 m a.s.l.), an Italian station located at about 800 m south-west from the town of Ny-Ålesund in the Svalbard archipelago. March to September is the operating period of the station. Lack of data in winter months is due to less maintenance of the instruments during the polar-night period. In the north-eastern direction toward the Ny-Ålesund research village, a clean area was established and motorized activity and other potentially contaminant activities were forbidden.^16^ The geographic location of GVB and the dominant winds ensure minimal anthropogenic contamination from local emissions while also capturing long-range transported pollution air masses.^5,16^ Aerosol size distributions were measured by a scanning mobility particle sizer (SMPS; TSI model 3034; 10.4–469.8 nm, 54 channels) and an aerodynamic particle sizer (APS; TSI model 3321; 0.542–19.81 μm, 51 channels). According to the specification sheets, typical single-channel uncertainty in aerosol number concentration measured by the SMPS and APS are ±20% and ±10%, respectively. The SMPS and APS are attached to the same inlet, which follows EUSAAR-ACTRIS protocol and is positioned about 4 m above the ground.^17^ The two instruments work with a time resolution of 10 min. Data are averaged over 1 h period and then reported at an hourly time resolution. In the present study, hourly data are averaged into daily data. The percentages of valid data for daily SMPS and APS from March to October 2015 are 81.2 and 93.9% (Table S1), respectively.

Daily PM_10_ filter samples were collected, using a Tecora SkyPost sequential sampler equipped with a PM_10_ sampling head, operating following the EN 12341 European protocol. The percentages of valid data for ions and metals from daily filter samples are 90.2 and 89.8% (Table S1), respectively. Inorganic anions (Cl^–^, Br^–^, NO_3_^–^, and SO_4_^2–^) and cations (Na^+^, NH_4_^+^, K^+^, Mg^2+^, and Ca^2+^) and selected organic anions [methanesulfonate (MSA) and oxalate] were analyzed using a three-Dionex ion chromatography system equipped with electrochemical-suppressed conductivity detectors.^18^ Analytic uncertainty is typically below 5%. The concentrations of metals (Al, As, Ba, Cd, Ce, Cr, Cu, Fe, La, Mn, Ni, Pb, Ti, V, and Zn) were determined by an ELEMENT2 (Thermo Fisher Scientific, Massachusetts, USA) inductively coupled plasma mass spectrometry (ICP–MS) instrument, a double-focusing magnetic sector field (SF) ICP-SF-MS coupled with a desolvation system provided with a microflow nebulizer.^19^

Meteorological parameters, including wind speed, wind direction, relative humidity, and ambient temperature, were recorded hourly at a height of 10 m (a.g.l.) on the Amundsen-Nobile Climate Change Tower in the neighborhood of the site.^20^ Surface net solar radiation, total cloud cover, total precipitation, snowfall, and boundary layer height were obtained from the Copernicus Climate Change Service (C3S), available at: https://cds.climate.copernicus.eu/cdsapp#!/dataset/reanalysis-era5-single-levels (last accesses: September 2021).

### 2.2 Source Apportionment

Source apportionment of the daily chemical composition data from filter samples complemented by SMPS and APS measurements was performed using the U.S. Environmental Protection Agency (US-EPA) positive matrix factorization (PMF) 5.0. Size distributions from SMPS and APS were not merged due to the absence of overlapping size bins from the two instruments, so the data with diameters <500 nm were mobility diameters and those with diameters >500 nm were aerodynamic diameters. Before PMF modeling, Na^+^ and Ca^2+^ were each apportioned to a sea salt (ss) fraction and a non-sea-salt (nss) fraction.^5^ In addition, SO_4_^2–^ was apportioned to sea salt (ss-SO_4_^2–^), mineral dust (mineral-SO_4_^2–^), biogenic (bio-SO_4_^2–^), and anthropogenic fractions (anthr-SO_4_^2–^) following previous studies.^5,16^ The particle volume concentrations (d*V*) at each size bin from SMPS and APS and mass concentrations of chemical species (ions: ss-Na+, nss-Na^+^, NH_4_^+^, K^+^, Mg^2+^, Cl^–^, NO_3_^–^, oxalate, MSA, Br^–^, ss-Ca^2+^, nss-Ca^2+^, ss-SO_4_^2–^, mineral-SO_4_^2–^, bio-SO_4_^2–^, and anthr-SO_4_^2–^; metals: Al, As, Ba, Cd, Ce, Cr, Cu, Fe, La, Mn, Ni, Pb, Ti, V, and Zn) were combined in a concentration matrix for the PMF model. To obtain an optimal solution with physical meaning, we applied relatively low uncertainties to chemical components but high uncertainties to SMPS and APS to make sure that factors resolved by the model were well separated mainly based on chemical composition (i.e., the model was driven by chemical composition and the particle size distributions followed). Details about settings for the PMF modeling and its limitation can be found in the Supporting Information, Text S1.

### 2.3 Back Trajectories and Concentration Weighted Trajectories

The Hybrid Single Particle Lagrangian Integrated Trajectory (HYSPLIT 5.0.0) model^21^ was used to calculate 7 day^5,22^ hourly backward trajectories arriving at an altitude of 100 m (a.g.l.) at the GVB from March to October 2015. Meteorological data (.gbl) from the National Centers for Environmental Prediction (NCEP) and the National Center for Atmospheric Research (NCAR) reanalysis data set were used to run the HYSPLIT model. Trajectories were clustered into six clusters based on an angle-based distance matrix, which is provide by “trajCluster” function in the “Openair” package in R.^23^ The back trajectories are divided into above or below the mixing layer height (ML, calculated by the HYSPLIT model). Following a previous study,^5^ daily surface types in the Northern Hemisphere are temporally allocated with the back trajectories. Each data point from the hourly back trajectories is labeled as (i) sea, (ii) sea ice, (iii) snow, (iv) land, or (v) above ML. In the present study, relative fractions of the surface types (i.e., air mass exposure to surface types) were calculated disregarding periods that the air mass spent above ML.

To investigate potential source regions leading to total particle concentration from each resolved source, the back trajectories were gridded to 1 ° × 1 ° grid cells and linked to particle concentrations by the following equation
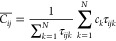
1where  is the concentration weighted by trajectories (CWT) at cell ij, *N* is the total number of trajectories, *c*_*k*_ is the concentration measured upon arrival of trajectory *k*, and τ_*ijk*_ is the residence time of trajectory *k* in grid cell (*i*,*j*). A high value of  means that air parcels passing over cell (*i*,*j*) could, on average, cause high concentrations at the sampling site. The CWT analysis was performed using the “trajLevel” function in the “Openair” package in R.^23^

### 2.4 Explainable Machine Learning Technique

Random forest (RF) modeling has been applied widely to reproduce air pollutant concentrations.^24^ Here, we built RF models using “RandomForestRegressor” function provided by “scikit-learn”^25^ in a python environment. PNs of the PMF-resolved factors are independent variables for the RF models. Explanatory variables include ambient temperature, boundary layer height, relative humidity, surface pressure, total cloud cover, snowfall, surface net solar radiation, total precipitation, wind speed, wind direction, air mass clusters, and relative fraction of the accumulated time for surface types (height below the mixing layer), that is, the time a back-trajectory air parcel spends over sea, sea ice, snow, and land. The hyper-parameters for the RF model were tuned using a function of “GridSearchCV” from the “scikit-learn” library. More details about the RF model and selection of the hyper-parameters are shown in the Supporting Information, Text S2. To investigate how specific processes drive the different aerosol factors, the SHAP approach was applied to interpret the RF models.^15^ SHAP is a game theoretic approach that is able to fairly distribute the anomaly of the total concentration among different parameters^14^
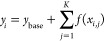
2where *x*_*i*,*j*_ is the value of feature *j* for sample *i*. *K* is the total number of different parameters. *f*(*x*_*i*,*j*_) is the SHAP value of feature *x*_*i*,*j*_, indicating the contribution of *x*_*i*,*j*_ to *y*_*i*_. The average value of the model predictions on all the samples is *y*_*base*_, baseline of the model. If *f*(*x*_*i*,*j*_) > 0 (*f*(*x*_*i*,*j*_) < 0), it means that the value of parameter *j* on sample *i* can increase (decrease) the aerosol concentration on sample *i* relative to the base value. The higher value of |*f*(*x*_*i*,*j*_)| represents a higher impact/importance of *x*_*i*,*j*_ on the corresponding measured aerosol concentration. The SHAP approach was implemented by the “shap” python package.^14^

## 3 Results and Discussion

### 3.1 Particle Number and Volume Size Distributions

**Figure 1:**
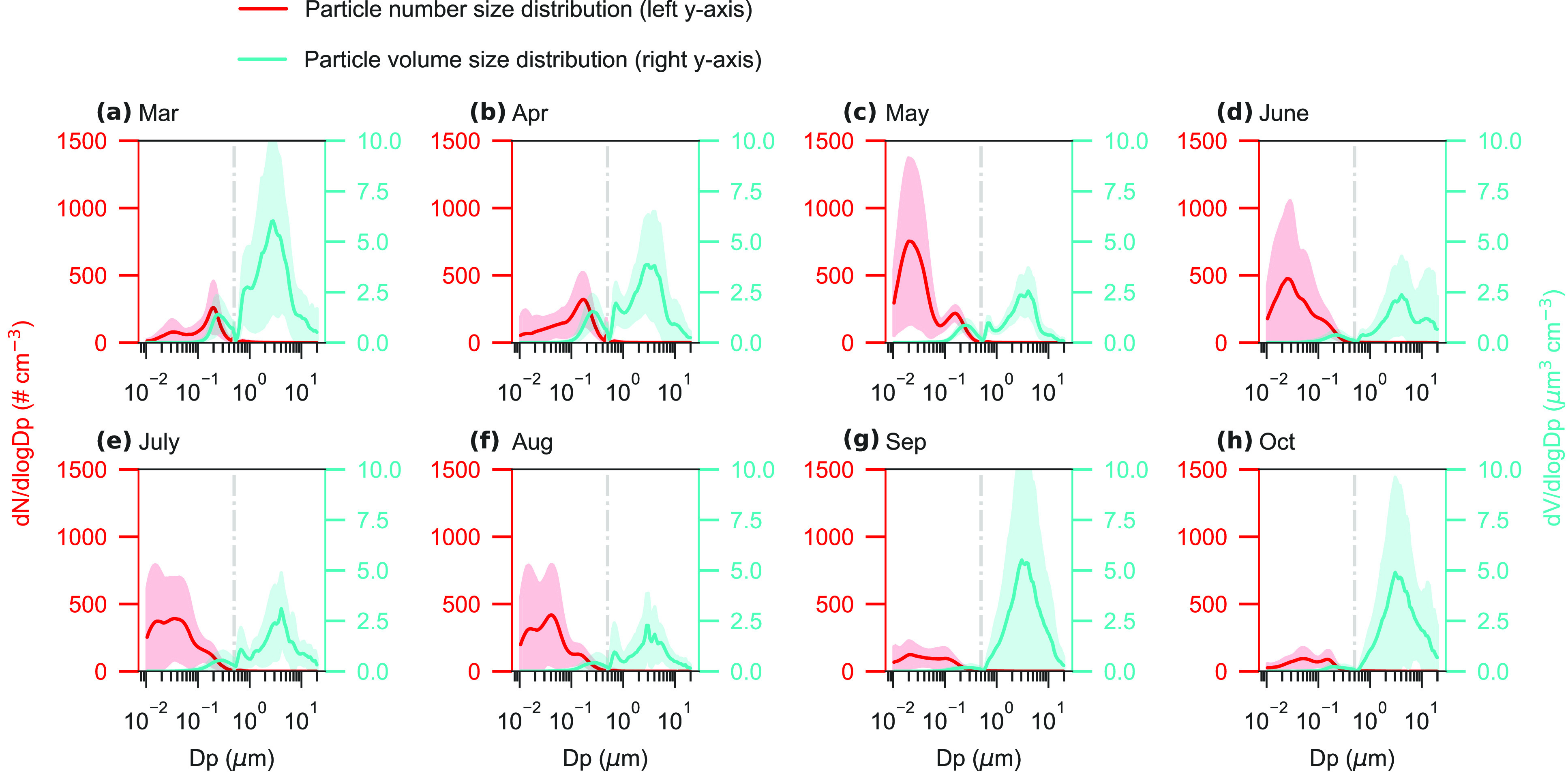
Monthly average particle number and volume size distributions. The measurements were conducted at the Gruvebadet Observatory in Svalbard from (a) March to (h) October 2015. The shaded area presents one standard deviation based on the daily data. Data for particle diameters of 10–470 nm are measured by an SMPS (TSI model 3034). Data for particle diameters of 0.5–20 μm are measured by an APS (TSI model 3321). The vertical dash-dot line in each subfigure denotes a diameter of 500 nm, which is between the largest diameter from SMPS (470 nm) and the lowest diameter from APS (0.5 μm). A valley at ∼500 nm in the volume distributions is due to the high measurement uncertainties (Supporting Information, Text S3) at the upper diameters of SMPS (up to 470 nm) and lower diameters of the APS (down to 0.5 μm).

The good data coverage (Table S1) of particles from 10 nm to 20 μm, though missing diameter range of 470–542 nm due to measurement limitation (Text S3), allows us to understand their monthly variations (Figure 1). Enhanced PN was observed from May to August with peaks in the nucleation mode, whereas enhanced volume concentration (PV) was observed in spring and autumn with peaks in the coarse mode (∼3 μm).

PNSD in March and April showed accumulation-mode peaks (dN/dlogDp) at ∼200 nm (262 ± 194 cm^–3^) and at ∼170 nm (321 ± 198 cm^–3^), respectively. However, particle volume (dV/dlogDp) exhibited a bimodal distribution in both the accumulation mode at ∼250 nm (1.4 ± 0.9 μm^3^ cm^–3^ in March and 1.5 ± 0.8 μm^3^ cm^–3^ in April) and the coarse mode at ∼3 μm (6.0 ± 4.6 μm^3^ cm^–3^ in March and 3.9 ± 2.4 μm^3^ cm^–3^ in April). In May, a bimodal pattern was observed for both PNSD and PVSD. For PNSD, in addition to a peak in the accumulation mode at ∼160 nm with 218 ± 56 cm^–3^, another much larger peak was found in the nucleation mode at ∼20 nm with 753 ± 618 cm^–3^, attributable to local NPF events^6^ with concurrent enhanced biogenic aerosols (MSA, Figure S1) in May.^26,27^

PVSD during June–August exhibited similar features; however, higher concentrations in the coarse mode of 10–20 μm were observed in June, likely due to dust-relevant spikes during June 11–13, 2015. For PNSD, there is a shift in the peak from ∼25 nm (475 ± 538 cm^–3^) in June to ∼40 nm (420 ± 374 cm^–3^) in August, with July (367 ± 326 cm^–3^ at ∼25 nm to 388 ± 309 cm^–3^ at ∼40 nm) in the middle showing a transition pattern with double peaks at ∼25 nm and ∼40 nm. PNs in September and October are lower but PVs—peaking in the coarse mode at ∼3 μm (5.5 ± 6.1 μm^3^ cm^–3^ in September and 4.9 ± 4.7 μm^3^ cm^–3^ in October)—were higher than the other months. On average, the coarse-model aerosols contributed ∼0.3% to particle number but ∼81.7% to particle volume concentrations.

### 3.2 Monthly Variation and Size Distribution of Different Aerosol Factors

A nine-factor solution was determined by PMF (Figure S2) as the optimal fit to both the chemical and PNSD/PVSD data (Figure S3 and Table S2). The PMF model successfully reproduced chemical species (Table S3) and total particle volume/number concentrations from SMPS and APS measurements, with high *R*^2^, and the scaled residuals are within the range of ±1 for all size bins (Figure S3). We identified the well-separated (Figure S4) factors as nucleation, biogenic, secondary, trace metals, anthropogenic, mineral dust (including mineral dust_1 and mineral dust_2), sea salt, and blowing snow aerosols (Table 1); their chemical and PNSD/PVSD profiles are shown in Figures S5 and S6, respectively. Details about the source identification of the nine factors are discussed in the Supporting Information, Text S4.

**Table 1:** Chemical Tracers, Dominating Diameter Ranges, Major Environmental Drivers, and Potential Source Regions for the Aerosol Factors^a^^,^^b^

aerosol factor	chemical tracers	dominating diameter range	contribution to PN/PV_10–20μm_	major drivers of monthly variation	potential source regions
F1: nucleation	N.A.	10.4–58.3 nm	52.0%/0.9%	solar radiation, boundary layer height, and wind speed	High Arctic and marginal ice zone
F2: biogenic	MSA, biogenic fraction of SO_4_^2–^	138.2–171.5 nm	7.5%/13.3%	solar radiation, surface pressure, and ambient temperature	High Arctic and marginal sea ice zones
F3: secondary	NO_3_^–^, NH_4_^+^ and oxalate	58.3–138.2 nm	21.9%/5.4%	ambient temperature, boundary layer height, and surface pressure	High Arctic and marginal sea ice zones, open ocean, northern Eurasia, northern Alaska, etc.
F5: mineral dust_1	Ca^2+^, non-sea salt fraction of Na^+^ and mineral fraction of SO_4_^2–^		4.6%/0.5%	surface pressure and ambient temperature	Arctic Archipelago, coastal region of Greenland, northern Eurasia, northern Alaska, and local dust
F6: anthropogenic	Pb, Cd, As, Br^–^, NH_4_^+^, and anthropogenic fraction of SO_4_^2–^	171.5 nm–0.835 μm	6.1%/8.1%	ambient temperature, surface pressure, and air mass cluster	Northern Eurasia
F7: sea salt	Cl^–^, Mg^2+^ and K^+^, sea-salt fraction of Na^+^ and Ca^2+^	0.835–6.264 μm	1.0%/36.2%	boundary layer height and air masses traveling over snow/open ocean/sea ice.	The Norwegian Sea, the Greenland Sea, the Baffin Bay, Greenland, Arctic Archipelago, etc.
F8: mineral dust_2	Al, Ba, Ce, Fe, La, Mn, Ti, and V		1.5%/6.7%	surface pressure and ambient temperature	Arctic Archipelago, coastal regions of Greenland, northern Eurasia, and northern Alaska
F9: blowing snow	Br^–^, sea salt-related species, high ratio of Br^–^/Na, and mineral dust-related trace metals	6.264–19.81 μm	3.2%/32.0%	boundary layer height and air masses traveling over sea ice/snow	Greenland, Arctic Archipelago, and northern Alaska

a The names of the regions are based on a previous study.^2^ Details about the source identification of the nine factors can be found in the Supporting Information, Text S4. The difference between mineral dust_1 and mineral_dust_2 is that mineral dust_1 may be of more local origin than mineral dust_2. The trace metal factor F4 is not shown in the table because its sources are not well defined. The potential source regions are based on CWT in Figure S9.

b Note: N.A.: not available.

**Figure 2:**
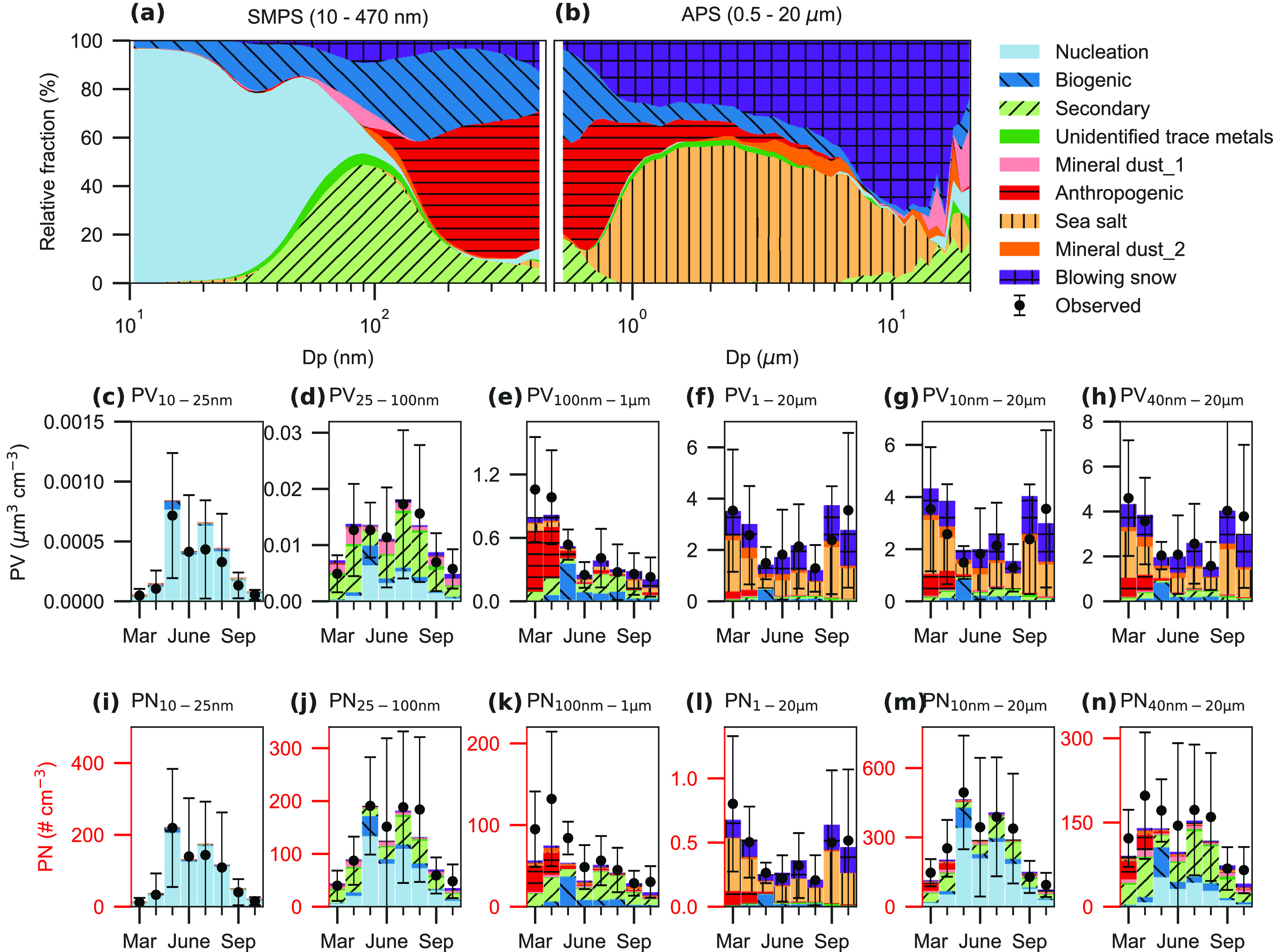
Size-resolved factor contributions and monthly variations in particle number (PN) and volume (PV) concentrations at different size bins. Size-resolved factor contributions for diameters measured by (a) SMPS (10–470 nm) and (b) APS (0.5–20 μm). Monthly variations in particle volume concentrations at diameter ranges of (c) 10–25 nm, (d) 25–100 nm, (e) 100 nm–1 μm, (f) 1–20 μm, (g) 10 nm–20 μm, and (h) 40 nm–20 μm. Monthly variations in PNs at diameter ranges of (i) 10–25 nm, (j) 25–100 nm, (k) 100 nm–1 μm, (l) 1–20 μm, (m) 10 nm–20 μm, and (n) 40 nm–20 μm. The observed concentrations are denoted by a black dot with one standard error bar.

Figure 2 illustrates size-resolved factor contributions and monthly variations in PN and PV in different size bins. Nucleation, secondary, biogenic, anthropogenic, sea salt, and blowing snow aerosols dominated particle diameters of 10.4–58.3, 58.3–138.2, 138.2–171.5, 171.5 nm–0.835, 0.835–6.264, and 6.264–19.81μm, respectively. The results suggest that the monthly variation in particles is highly size and source dependent. The total particles show a clear monthly variation—a summer maximum in particle number due to enhanced nucleation and a spring/autumn maximum in particle volume caused by enhanced sea salt, rather than the Arctic haze identified in the nearby Zeppelin station.^2,28^ This is likely due to a smaller impact of sea salt at Zeppelin (475 m, a.s.l.) than at GVB (61 m, a.s.l.).

Distinct monthly variations at different size ranges for PN and PV can be explained by the seasonality of aerosol source contributions (Data S1). Monthly variation of PN_10–25 nm_ and PN_25–100nm_ was dominated by the nucleation factor (Figure 2), which peaked in summer (Figure S7) with monthly average contributions of 91.3 ± 6.4% (78.8% in March—97.6% in July) and 46.3 ± 20.0% (16.0% in March—70.1% in May), respectively. At least some particles smaller than 30 nm in diameter can be activated as CCN in the Arctic;^29^ therefore, nucleation has a high potential to influence cloud formation in the summertime Arctic. Secondary aerosols made a more significant contribution (28.8 ± 9.4%) to PV_25–100 nm_ than the nucleation factor (13.9 ± 9.1%), as secondary aerosols dominated the larger ultrafine particle bins (62.6–100 nm) (Figure 2).

Monthly variation of PN_100nm–1μm_ and PV_100nm–1μm_ was driven by multiple aerosol factors (Figure 2), including anthropogenic, biogenic, and secondary aerosols. In March and April, Arctic haze contributed the most to both PN_100nm–1μm_ (57.4 and 36.6%, respectively) and PV_100nm–1μm_ (72.2 and 59.3%, respectively). In May, biogenic aerosol was the main source with average contributions of 66.0% to PN_100nm–1μm_ and 67.1% to PV_100nm–1μm_, primarily due to high biogenic emissions, such as high dimethylsulfide (DMS) concentrations observed in Svalbard in May 2015.^27^ However, secondary aerosols generally dominated PN_100nm–1μm_ (52.2 ± 14.2%) and PV_100nm–1μm_ (32.4 ± 13.9%) during June–October. These results demonstrate the complexity of the sources and processes influencing the accumulation-mode aerosol.

For coarse-mode particles, sea salt aerosol dominated both PV_1–20μm_ (41.8 ± 9.3%) and PN_1–20μm_ (49.9 ± 9.6%) in almost all seasons, which is consistent with the occurrence frequency-based results.^5^ Higher sea-salt concentrations were observed in spring/autumn than in summer (Figures S7 and S8). The peak of sea salt concentration in September is likely due to the higher fraction of air mass traveling over open ocean compared to the other months (Figure S8a). However, the second peak in March is more difficult to explain. A possible source is from pack ice^30^ and snow,^31^ considering the high wind speeds associated with air masses traveling over sea ice and snow (Figure S8).

Sea salt and blowing snow contributed 36.2 ± 9.5% (20.1% in May—49.5% in September) and 32.0 ± 9.8% (18.6% in May—39.5% in July) to the overall PV_10nm–20μm_, respectively. Although sea salt and blowing snow contributed notably to super-micrometer particles in both number (49.4 ± 9.6% and 30.3 ± 8.4, respectively) and volume (41.8 ± 9.3 and 35.8 ± 8.6%, respectively), they contributed very little to sub-micrometer and total particle number (Figure 2). These results suggested that sea salt aerosol might be less important than previously assumed given its small contribution to PN.

Mineral dust (the sum of mineral dust_1 and mineral dust_2) contributed 7.8 ± 4.2% (2.8% in August—15.0% in June) to the overall PV_10nm–20μm_, which is comparable to 6.9 ± 5.5% based on an occurrence frequency-based study.^5^ Nucleation makes a major contribution (52 ± 22%, 15.6% in March—73.4% in May) to overall PN_10nm–20μm_. Compared to nucleation, the contributions from the other factors to PN_10nm–20μm_ are much smaller: 21.9 ± 10.1% from secondary, 7.5 ± 5.1% from biogenic, 6.1 ± 4.1% from mineral dust (4.6 ± 3.1% from mineral dust_1 and 1.5 ± 1.1% from mineral dust_2), 6.1 ± 10.6% from anthropogenic, 3.2 ± 3.3% from blowing snow, 2.2 ± 1.5% from trace metal factor, and 1.0 ± 0.9% from sea salt. The sources of the trace metal factor are not clear (Supporting Information, Text S4), but it contributed only 1.8 ± 0.8% to PV_10nm–20μm_ and 2.2 ± 1.5% to PN_10nm–20μm._

### 3.3 Potential Source Regions of the Aerosol Factors

Potential source regions of the aerosol factors in spring (including March–May), summer (including June–August), and autumn (including September–October, no valid data in November) seasons are shown by CWT in Figure S9. The anthropogenic factor appears to be associated with air masses arriving from northern Eurasia, particularly during the Arctic haze period (Figure S9). Snow-covered regions (e.g., Greenland and Arctic Archipelago) were potential source regions for both sea salt and blowing snow in spring and autumn, which is consistent with the enhanced sea salt and blowing snow aerosols in these months (Figure S7). Dust particles in Svalbard are associated with air masses (Figure S9) from (i) northern Eurasia in spring, (ii) northern Alaska in summer, (iii) the Arctic Archipelago and coastal regions of Greenland in autumn, and (iv) local sources (e.g., glacial outwash sediments in the summertime^32^), which have been identified as potential high latitude dust source regions.^33^

The potential source-region map for secondary aerosol shows that the peak in summer and that in spring are related to different source regions (Figure S9), with the former linked to open ocean (i.e., natural source) and the latter to Arctic continental (i.e., anthropogenic source) regions. Unlike the above aerosol factors, high CWT values for nucleation and biogenic aerosols were found in the High Arctic and marginal ice zone in summer (i.e., June–August) and spring (primarily in May), respectively, implying enhanced emission or formation from sea ice.^29,44^ This is consistent with sea ice being an important source of alkylamines and the precursors of sulfur and iodine oxoacids, all of which can efficiently participate in NPF processes.^29,34,35^ However, the High Arctic and marginal ice zone did not contribute much to nucleation aerosol in spring and autumn (Figure S9), implying that sea ice exposure alone is not sufficient to promote the nucleation processes.

### 3.4 Environmental Drivers of the Aerosol Factors

**Figure 3:**
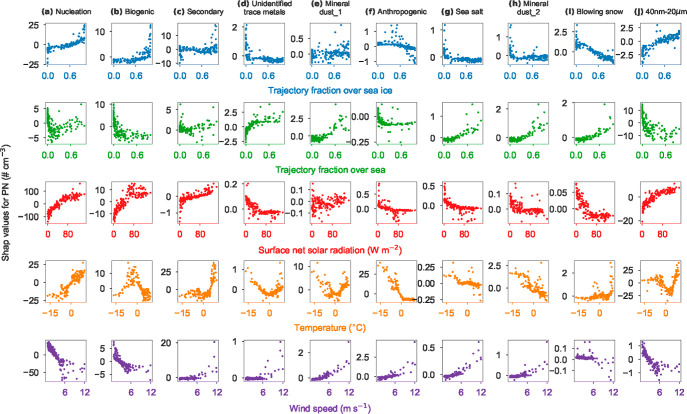
SHAP responses of the resolved aerosol factors to different parameters. The aerosol factors from left panel to right panel are (a) nucleation aerosol, (b) biogenic aerosol, (c) secondary aerosol, (d) unidentified trace metals, (e) mineral dust_1, (f) anthropogenic aerosol, (g) sea salt, (h) mineral dust_2, (i) blowing snow, and (j) particles in the diameter range of 40 nm–20 μm. The trajectory fractions of sea and sea ice represent relative fractions of the accumulated time for air masses traveling over the surface types without considering air masses above the mixing layer. The responses to additional parameters are shown in Figure S13. Note that different scales are applied to the *y*-axis for each subfigure.

Understanding potential drivers of the aerosol factors rather than total aerosol is crucial as each aerosol factor responds to changes in environmental processes differently. The relationships between each aerosol factor identified in this work and meteorological/environmental variable are shown in Figure S10. Sea salt, blowing snow, mineral dust, and anthropogenic aerosols are often associated with high wind speed, whereas nucleation, biogenic, and secondary aerosols are associated with high solar radiation. In addition, low relative humidity appears to facilitate resuspended dust, and anthropogenic aerosols are mainly present in cold seasons, and enhanced sea salt and blowing snow aerosols are associated with high wind speed and high boundary layer height. The random forest models are able to reproduce the aerosol factors (Figure S11); therefore, the responses of each of the meteorological parameters on every prediction for the nine aerosol factors were quantitatively investigated (Figures 3 and S12–S14) by the SHAP approach. None of the meteorological parameters have the same impacts on all the aerosol factors, further highlighting the importance of breakdown of total aerosol into different aerosol factors.

For nucleation aerosol, the most important parameter with positive responses is solar radiation, followed by temperature and sea ice exposure (i.e., the fraction of the accumulated time for air masses traveling over sea ice) (Figure S12), consistent with their key roles in photochemistry.^36,37^ Temperature-dependent gas precursor emissions and high-temperature days coincident with high solar radiation may potentially explain the positive response of nucleation aerosol to temperature. A sea ice trajectory fraction of 0 was observed as an activation threshold for nucleation aerosol (Figure 3), suggesting that sea ice is an important source region of precursors for NPF.^29,34,38^ Both enhanced sea ice exposure and high solar radiation appear to be key drivers of nucleation aerosol number (Figures 3, S12 and S14). Boundary layer height and wind speed also appear to be significant drivers for nucleation aerosol, though with a negative response (Figures 3 and S13 and S14). Enhancement of nucleation aerosol in July may be associated with low boundary layer height and low wind speed (Figures S8 and S14), indicating the importance of local NPF. This is consistent with results from the nearby Zeppelin, Svalbard.^44^ Overall, solar radiation, boundary layer height, and wind speed are the main drivers governing the monthly variation in nucleation aerosol (Figure S14).

We found that solar radiation (i.e., key factor for free radicals^27^ and aerosol precursor emissions), surface pressure (i.e., air mass transport^26^), and temperature are the most important parameters (Figure S12) responsible for the monthly variation in biogenic aerosol (Figure S14). It is known that MSA concentrations are related to temperature both directly through DMS oxidation^39,40^ and indirectly through emissions from melting sea ice.^26^ Our results show that temperature influences the monthly variation in biogenic aerosol with a nonlinear response (Figure 3)—the concentration of biogenic aerosol increased with temperature up to ∼0 °C and then decreased with temperature above it. The inflection points at ∼0 °C may indicate a key role of sea ice melting, rather than DMS oxidation for the biogenic aerosol.

The monthly variation in secondary aerosol was mainly governed by temperature, boundary layer height, and surface pressure (Figures S12 and S14). The first and second peaks in the monthly variation (Figure S7) of secondary aerosol were mainly attributed to low surface pressure (i.e., air mass transport) in April and high temperature (i.e., photochemical oxidation) during July–August, respectively (Figures 3, S8, S13, and S14).

The drivers of the trace metal factor are more difficult to determine (Figures 3 and S12–S14). More air masses traveling over open ocean and continental regions during August–October compared to the other months (Figure S8) may have led to enhanced contributions from both natural and anthropogenic (e.g., industrial and shipping) sources.

The monthly variations in both mineral dust factors were largely driven by surface pressure and ambient temperature (Figures S12 and S14). Low humidity and high wind speed facilitated dust emissions (Figures 3 and S12 and S13). The differences between the two types of mineral dust are: (i) high temperature-driven enhancement of mineral dust_1 in June and July (Figure S14) and (ii) more contributions from mineral dust_1 to particles larger than 10 μm relative to mineral dust_2 (Figure 2). Thus, mineral dust_1 may be of more local origin than mineral dust_2. Mineral dust_1 showed a nonlinear response to temperature with the inflection point at ∼0 °C (Figure 3), the melting temperature for snow/ice on land, indicating that the snow/ice coverage on land regulates resuspended dust.

The monthly variation in anthropogenic factor was largely driven by temperature with small contributions from surface pressure and air mass cluster (Figure S14). This is because meteorological conditions during cold seasons are conducive to long-range transport of anthropogenic pollution from northern Eurasia (Figure S9), leading to Arctic haze.^4^ An inflection point at ∼0 °C was observed (Figure 3), indicating that ∼0 °C might be a threshold for long-range transported anthropogenic aerosols.

Boundary layer height and air masses traveling over sea ice and snow were the common parameters controlling the monthly variations (Figures S12 and S14) of sea salt and blowing snow factors. However, wind speed and air masses traveling over open ocean were more important for the sea salt factor than for the blowing snow factor (Figures S12 and S14). In addition, large snowfall could facilitate blowing snow aerosol (Figure S13).

As expected, the drivers for total aerosol in number and volume (Figure S14) are similar to those for nucleation aerosol and sea salt aerosol, respectively.

### 3.5 Implications

This study has quantified sources of both particle number and volume from 10 nm to 20 μm in Svalbard, the first source apportionment study of this kind at the high latitude. A range of aerosol factors were identified, including nucleation, secondary, trace metal, biogenic, anthropogenic, sea salt, and blowing snow aerosols, though they can be overlapped. Table 1 summarizes main results for the identified aerosol sources. The PNSD profiles (Data S2) for the nine aerosol sources will be useful for future source apportionment studies at the high latitude. Sea salt and blowing snow aerosols contributed substantially to total particles in mass/volume; however, their contributions to sub-micrometer and total particles in number are small. Since particle numbers with diameter greater than 40 nm are highly correlated to number of CCN in the Arctic,^4,41^ we presented results for PN_40nm–20μm_ in Figures 2 and 3. The results suggested that secondary and nucleation-derived aerosols dominated potential CCN (PN_40nm–20μm_ as a proxy) with monthly average contributions of 40.4 ± 13.1 and 20.2 ± 12.8%, respectively. Considering recent evidence that NPF-derived particles can be activated as CCN smaller than 30 nm,^29^ the potential contribution from nucleation aerosol is probably even greater.

By an explainable machine learning technique, we showed a highly nonlinear and variable response of aerosols of different origins to environmental parameters and thus ongoing Arctic warming. In particular:

1 *Nucleation*: with the shift from a polar to a marine environment and change in Arctic cloud properties, the response of nucleation aerosol to Arctic warming is highly uncertain. An increase in frequency of nucleation events was found to be correlated with decrease in sea ice extent, which might be related to enhanced organic nitrogen/amine emissions from melting sea ice regions.^8,35^ However, the much greater impact of solar radiation on nucleation than sea ice exposure (Figures 3 and S14) should be considered for better understanding the direct and indirect responses of nucleation to sea ice melting.
2 *Biogenic and secondary aerosol:* Our results suggest that biogenic aerosol is likely to increase as a result of sea ice melting in a warming Arctic but decreases when the average temperature is above ∼0 °C. Such a nonlinear response to temperature may explain the inter-annual variation in biogenic aerosol^42^ and affect nucleation processes^43^ because the biogenic factor is a potential source of nucleation aerosols (Figure 2a). The response of secondary aerosol to Arctic warming is more complicated because it has both anthropogenic and natural origins.^12^ A decrease in biogenic aerosol above 0 °C may be counterbalanced by an increase in secondary aerosol.
3 *Mineral dust*: The nonlinear response of local dust to temperature with an inflection point at ∼0 °C suggests that both the seasonality and emissions will change in a warming Arctic.
4 *Sea salt and blowing snow*: Sea salt aerosol is likely to increase due to both more air masses traveling over ocean and strengthening of surface winds over the Arctic Ocean as a result of sea ice losses.^22,44^ Blowing snow aerosol is likely becoming less important compared to sea salt due to the melting snow and reduced snowfall in a warmer Arctic.
5 *CCN*: Monthly variation in potential CCN is driven by multiple meteorological parameters (Figure S14). Boundary layer height, temperature, and air mass cluster are the key parameters (Figure S12), and sea ice is an important source region for potential CCN (Figure 3j). Potential CCN responded to temperature nonlinearly with an inflection point at ∼0 °C.

In summary, this study presents a framework of a receptor model coupled with machine learning to understand sources and drivers of wide-range particles at a high Arctic site, and the results show that each aerosol factor will be affected by environmental change differently, highlighting the complexity of Arctic aerosol. Further measurements in winter seasons and at other Arctic sites will be needed to develop an Arctic-wide understanding.^7,12^ Our findings point to the key aerosol processes to focus on when quantifying the sensitivity of aerosol sources and responses to environmental drivers.

## Supporting Information Available

Details of the measurement data and details about parameter settings, performance, output, and interpretation for the PMF, CWT, RF, and SHAP modeling (PDF)
Source contributions of the nine factors to observed aerosols at different size bins (XLSX)
Fractional particle number size distribution profile for each aerosol factor (XLSX)
