# Supplementary material for: Understanding Sources and Drivers of Size-Resolved Aerosol in the High Arctic Islands of Svalbard Using a Receptor Model Coupled with Machine Learning
Source: Environ Sci Technol. 2022 Jul 25;56(16):11189–98. doi: 10.1021/acs.est.1c07796 (PMC9386907; doi:10.1021/acs.est.1c07796)
Supplement: Supplementary file 1 — es1c07796_si_001.pdf [file es1c07796_si_001.pdf]

Supporting Information *for*

Understanding sources and drivers of size-resolved aerosol in the high Arctic Islands of Svalbard using a receptor model coupled with machine learning

Congbo Song[1*], Silvia Becagli[2,3], David C. S. Beddows[4], James Brean[1], Jo Browse[5], Qili Dai[6], Manuel Dall'Osto[7], Valerio Ferracci[8], Roy M. Harrison[1,a], Neil Harris[8], Weijun Li[9], Anna E. Jones[10], Amélie Kirchgäßner[10], Agung Ghani Kramawijaya[1], Alexander Kurganskiy[5], Angelo Lupi[11], Mauro Mazzola[11], Mirko Severi[2,3], Rita Traversi[2,3] and Zongbo Shi[1*]

[1]School of Geography, Earth and Environment Sciences, University of Birmingham, Birmingham B15 2TT, UK

[2]Department of Chemistry "Ugo Schiff", University of Florence, Via della Lastruccia 3, 50019 Sesto Fiorentino, Italy

[3]National Research Council of Italy, Institute of Polar Sciences (CNR-ISP), Via Torino 155, 30172 Venice-Mestre, Italy

[4]National Centre for Atmospheric Science (NCAS), School of Geography, Earth and Environmental Sciences, University of Birmingham, Birmingham B15 2TT, UK

[5]Centre for Geography and Environmental Science, University of Exeter, Penryn TR10 9FE, UK

[6]State Environmental Protection Key Laboratory of Urban Ambient Air Particulate Matter Pollution Prevention and Control, College of Environmental Science and Engineering, Nankai University, Tianjin, 300350, China

[7]Institute of Marine Science, Consejo Superior de Investigaciones Científicas (CSIC), Barcelona 08003, Spain

[8]Centre for Environmental and Agricultural Informatics, School of Water, Energy & Environment, Cranfield University, College Road, Cranfield MK43 0AL, Bedfordshire, UK

[9]Department of Atmospheric Sciences, School of Earth Sciences, Zhejiang University, Hangzhou 310027, China

[10]British Antarctic Survey, Natural Environment Research Council, Cambridge CB3 0ET, U.K.

[11]National Research Council of Italy, Institute of Polar Sciences (CNR-ISP), Via P. Gobetti 101, 40129 Bologna, Italy

[a]also at: Department of Environmental Sciences/Center of Excellence in Environmental Studies, King Abdulaziz University, P.O. Box 80203, Jeddah, 21589, Saudi Arabia

Correspondence: Congbo Song (c.song.1@bham.ac.uk) and Zongbo Shi (z.shi@bham.ac.uk)

This Supporting Information includes 30 pages, 4 texts, 14 figures and 3 tables.

Text S1. Settings for PMF modelling

Positive matrix factorization (PMF) is an advanced receptor model that decomposes a matrix of sample data (X) into two matrices, the factor contribution matrix (G) and the source profile matrix (F), based on observations at the sampling site. The PMF model can be expressed as follows:[1]

$x_{ij} = \sum_{k=1}^{p} g_{ik} \cdot f_{kj} + e_{ij}$ (eq. S1)

where x_{ij} is the measured concentration of the jth species in the ith sample, f_{kj} is the concentration of the jth species from factor k, g_{ik} is the contribution of the factor k to the ith sample and e_{ij} is the residual matrix for the jth species in the ith sample, and p is the total number of independent factors.

The parameters ($g_{ik}$and f_{kj}) are constrained to nonnegative values. The task of PMF is to calculate the minimum value Q, as follows:

$Q(E) = \sum_{i=1}^{n} \sum_{k=1}^{m} \left(\frac{e_{ij}}{\sigma_{ij}}\right)^2$ (eq. S2)

where σ_{ij} is the uncertainty in the jth species for the ith sample.

The particle volume concentrations (dV) at each size bin from SMPS (10.4 – 469.8 nm, 54 bins) and APS (0.542 – 19.81 µm, 51 bins), total particle volume concentration (i.e., 10 nm - 20 µm, $PV_{10nm-20\mu m}$) from combined SMPS and APS, and mass concentrations of chemical species (ions: ss-Na+, nss-Na^+, NH_4^+, K^+, Mg^{2+}, Cl^-, NO_3^-, Oxalate, MSA, Br^-, ss-Ca^{2+}, nss-Ca^{2+}, ss-SO_4^{2-}, mineral-SO_4^{2-}, bio-SO_4^{2-} and anthr-SO_4^{2-}; metals: Al, As, Ba, Cd, Ce, Cr, Cu, Fe, La, Mn, Ni, Pb, Ti, V and Zn) are combined in a concentration matrix for the PMF model. All missing data of input variables were replaced by their median value and the corresponding uncertainty was set to three times that value. For selected chemical species, concentrations below method detection limit (MDL) values were replaced by half of their MDL values. The corresponding uncertainties of these values were set at five sixths of the MDL values. Uncertainties for SMPS and APS were estimated using the following equation:[2]

$\sigma_{i,j} = a_j(N_{i,j} + \overline{N_j})$ (eq. S3)

where $\sigma_{i,j}$ and $N_{i,j}$ are the estimated measurement error and measured concentration for the jth species of ith sample, respectively. $\overline{N_j}$ is the arithmetic mean of the measured concentration for species j. a is an empirical constant. Here we use an empirical constant a = 0.01 following a previous study.[2] High uncertainties are applied to particle volume concentrations at the lowest and highest bins of SMPS and APS because of their elevated measurement error. The first lowest and highest size bin were assigned with multiplier a = 3, and the second lowest size bins and highest bin were assigned with multiplier of a = 2.5. For subsequent lower and higher size bins, the multiplier was incrementally reduced by 0.5 until it reaches a = 1. Total particle volume concentration (i.e., 10 nm - 20 µm, $PV_{10nm-20\mu m}$) from combined SMPS and APS measurements was assigned a multiplier a = 5.

The overall uncertainties were estimated by the following equation.[2]

$\delta_{i,j} = \sigma_{i,j} + C_3 \times N_{i,j}$ (eq. S4)

where $\sigma_{i,j}$ was estimated from equation (3) and C_3 is a constant. Different values of C_3 between 0.1 and 0.6 were tested to achieve the best calculated value for objective function Q (closest to the theoretical value, i.e., Q(true)/Qexp is closest to 1, see **Fig. S2**). A value of 0.45

was assigned to C_3 for all size bins of SMPS and APS as well as the total $PV_{10nm-20\mu m}$, while C_3 was set to 0.6 for chemical species.

Variables of all the chemical species were assigned as "strong", all the other variables were assigned as "weak" (the uncertainty $\delta_{i,j}$ will be $3\times\ \delta_{i,j}$ by setting them as "weak") and $PV_{10nm-20\mu m}$ was set as "total variable". Thus, the overall uncertainty multiplier for size bins is larger than that for chemical species, i.e., 0.45× 3 for size bins versus 0.6 for chemical species. The optimal PMF solution was chosen from four to thirteen factors by examining the physically meaningful profiles (**Fig. S2**) as well as model performance (e.g., R^2 for the variables and the distributions of scaled residuals, see **Fig. S3**). The effects of measurement error and rotation ambiguity on the optimal solution were examined by error estimation methods of bootstrap and displacement (DISP) in EPA PMF 5.0 (**Table S2**).

There are a few output files after running the EPA PMF 5.0 using the above settings, such as time series of factor concentrations with average value of 1 (i.e., $|G| = 1$), and factor profiles including concentration of species, % of species sum and % of total variable. The total $PV_{10nm-20\mu m}$ for each factor was apportioned to each size bin from SMPS (10.4 – 469.8 nm) and APS (0.542 – 19.81 μm) by using fractional profile of total $PV_{10nm-20\mu m}$ in each factor. Then volume concentrations in each size bin measured by SMPS were applied a scaling constant, obtained by regressing the measured $PV_{10-470nm}$ against the modelled $PV_{10nm-470nm}$. Same approach was applied to each size bin measured by APS. The particle number size distributions for each factor can then be converted from the particle volume size distributions. Our PMF modelling used a one-step method through the combination of two heterogeneous data, particle volume concentration (10 nm – 20 μm) and chemical data (PM_{10} (<10 μm)). Although they have different units, volume concentrations can be converted to mass concentrations by multiplying a factor of particle density, which means that the two datasets have equivalent homogeneous units.

There are a few limitations of the above one-step method: 1) the source apportionment results of the particle size distributions are largely dependent on the performance of the source apportionment of the chemical data (i.e., PM_{10}). 2) the PM_{10} chemical data (mass-based) does not provide chemical information of coarse mode particles in 10 – 20 μm though PV_{10} and PV_{20} show similar temporal variations and have the same correlation with PM_{10}, both have the R^2 of 0.78. 3) the OC and EC concentrations were not included in the PMF analysis due to data availability.

The reason we didn't drop the diameters of 10-20 μm is that adding more variables, even gaseous pollutants or meteorological parameters, can help reduce rotational ambiguity in PMF solutions.[4,5] In addition, the data with diameters of 10 – 20 μm have a negligible impact on the PMF results as high uncertainties were applied to these diameter ranges.

Text S2. RF model and selection of hyper-parameters

The important parameters for the random forest regressor ("RandomForestRegressor" function) from the "scikit-learn" include:

1) "n_estimators": the number of trees in the forest, default = 100. In general, the more trees the less likely the algorithm will be overfitting. The lower the number of trees, the closer the model is to a decision tree, with a restricted feature set. In general, the larger the

number of trees, the stabler the RF is. However, a large number of trees can make the algorithm too slow. The practical values for "n_estimators" are in the range of 300 and 500.

2) "max_features": the number of features to consider when looking for the best split. Default = "auto" (i.e., max_features = n_features). This determines how many features each tree is randomly assigned. The smaller, the less likely to overfit, but too small will start to introduce under fitting. The common options for this parameter are "auto" (max_features = n_features), "sqrt" (max_features = sqrt(n_features)), "log2" (max_features = log2(n_features)).

3) "max_depth": The maximum depth of the tree, default=None. If None, then nodes are expanded until all leaves are pure or until all leaves contain less than min_samples_split samples. Lowering the maximum depth can reduce the complexity of the learned models and lowering overfitting risk. Usually, try small depth of 5-10 and increase the value depending on the results.

4) "min_samples_leaf": the minimum number of samples required to be at a leaf node, default=1. This parameter has a similar effect to the "max_depth" parameter, it means that the branch will stop splitting once the leaves have that number of samples each. Usually, we try value greater than 1 (start from 2) to lower the overfitting risk.

5) "bootstrap": Whether bootstrap samples are used when building trees. If False, the whole dataset is used to build each tree.

6) "max_samples": If bootstrap is True, the number of samples to draw from the whole dataset to train each base estimator. If float, then draw max_samples * X.shape[0] samples. Thus, "max_samples" should be in the interval (0.0, 1.0].

The hyper-parameters for the RF model were tuned using a function of "GridSearchCV" from the "scikit-learn" library. The parameter ranges for running the "GridSearchCV" are:
"n_estimators": [100, 200, 300, 400, 500]
"max_features": ['auto', 'sqrt', 'log2']
"max_depth": [5, 6, 7, 8, 9, 10]
"min_samples_leaf": [2, 3, 4]
"bootstrap": [True, False]
"max_samples": [0.25, 0.5, 0.75]
The codes as follows:
"

```
from sklearn.model_selection import RandomizedSearchCV
from sklearn.ensemble import RandomForestRegressor
features = [ 'Land', 'Sea', 'Sea Ice', 'Snow', 'T', 'BLH', 'RH', 'SP', 'TCC', 'SF',
        'SSR', 'TP', 'WS', 'WD', 'Cluster']
X = df[features]
Y = df['PN10nm-20µm']
X_train, X_test, Y_train, Y_test = train_test_split(X, Y, test_size = 0.25, random_state=42)
```

```
random_grid = {
   'max_depth': [5,6,7,8,9,10],
   'max_features': ['auto', 'sqrt','log2'],
   'min_samples_leaf': [2,3,4],
   'n_estimators': [100, 200, 300, 400,500],
   'bootstrap': [True, False],
   'max_samples': [0.25,0.5,0.75]
}
rf = RandomForestRegressor()
rf_random = RandomizedSearchCV(estimator = rf, param_distributions = random_grid, n_iter = 100, cv = 3, verbose=2, random_state=42, n_jobs = -1)
rf_random.fit(X_train, Y_train)
"
```

The most important arguments in RandomizedSearchCV are n_iter, which controls the number of different combinations to try, and cv which is the number of folds to use for cross validation (we use 100 and 3 respectively).

The best parameters from fitting the random search can be viewed by running:

```
rf_random.best_params_
```

Returning results:

```
{'n_estimators': 400,
 'min_samples_leaf': 2,
 'max_samples': 0.75,
 'max_features': 'sqrt',
 'max_depth': 10,
 'bootstrap': False}
```

Thus, the hyper-parameters for the RF model are determined as above. The accuracy (1 – (Number of misclassified samples / Total number of samples)) of the RF model with the selected parameters is 72.88%. The coefficient of determination (R^2) of the RF model is 0.75. The performance of the RF model is much better than a multiple linear regression model (R^2 of 0.31) using the same dataset.

Text S3. Missing diameter ranges of total particle concentration

In this study, the measured diameter ranges of SMPS and APS are 10.4 – 469.8 nm of mobility diameters and 0.542 – 19.81 µm of aerodynamic diameters, respectively. Size distributions from SMPS and APS were not merged in our study due to the absence of overlapping size bins from the two instruments and high measurement uncertainties at the upper diameters of SMPS (up to 470nm) and lower diameters of the APS (down to 0.5µm). Missing size range between 10 nm and 20 µm from combined SMPS and APS is 470 – 542 nm. Note that $PN_{10nm\text{-}20\mu m}$/ $PV_{10nm\text{-}20\mu m}$ in the present study mean the sum of particle number/volume concentrations at diameter ranges of 10.4 – 469.8 nm mobility diameters measured by SMPS and 0.542 – 19.81 µm aerodynamic diameters measured by APS, without the inclusion of particles at 470 – 542 nm.

The high measurement uncertainties result in a valley at ~500 nm in the volume distributions (Figure 1). However, the measurement uncertainties and missing data from 470 – 542 nm may have negligible impacts on the total particle number concentrations (‘$PN_{10nm-20\mu m}$’) since particle number concentrations (dN/dlogDp) are close to 0 for particle at diameters of ˜500 nm (SMPS: ~13±1 cm^{-3} at 470 nm, APS: ~1.3±0.1 cm^{-3} at 0.542 μm, see Figure 1). It is challenging to quantify the impacts of the uncertainties since there are no reference/reliable concentrations for particles at ˜500 nm. Future measurements/studies are needed to measure particle size distributions with mobility diameter up to 1 μm to be better merged with aerodynamic particle size distributions by APS.

Text S4. Source identification of the nine factors resolved by PMF

F1: No obvious chemical signature (**Fig. S5a**) but dominated nucleation/Aitken mode with diameters of 10.4 – 58.3 nm (**Fig. 2**). The PNSD shows a unimodal distribution with a peak at ~20nm (**Fig. S6a**). High concentrations of F1 were observed from May to August (**Fig. S7a**) with high solar radiation (**Fig. S8b**) and high frequency of NPF events in Svalbard.[6,7] Thus, F1 is identified as a nucleation factor.

F2: High contributions (>97.3%) to MSA and biogenic fraction of SO_4^{2-} (bio-SO_4^{2-}) (**Fig. S5b**). F2 dominated particles with diameters of 138.2 – 171.5 nm. A bimodal distribution at peaks of Aitken mode (i.e., 25 – 100 nm) at 30 nm and accumulation mode at 150 nm was observed in the PNSD profile (**Fig. S6b**). The PVSD has two sub-micrometer modes (~0.3 and 0.7 μm) and one super-micrometer mode (~2 μm), which is consistent with that of MSA in summer.[8] A recent study also found that a fraction of MSA could occur in the coarse mode and is not entirely in the sub-micrometer fraction.[9] The monthly pattern observed for F2 (**Fig. S7b**) is similar to that of MSA and bio-SO_4^{2-} (**Fig. S1**). Daily concentrations of F2 are correlated to those of MSA with R^2 of 0.82. Thus, F2 is identified as a biogenic factor.

F3: High contributions to NO_3^- (43.4%), NH_4^+ (39.6%) and oxalate (71.3%) (**Fig. S5c**). The flat peak in PNSD profile for F3 extended from ~50 nm to ~150 nm, whereas the PVSD is dominated by the accumulation mode (**Fig. S6c**). F3 dominated Aitken-mode particles with diameters of 58.3 – 138.2 nm (**Fig. 2a**). Monthly variation of median volume concentration of F3 (**Fig. S7c**) is similar to that of NO_3^-, NH_4^+ and oxalate (**Fig. S1**), which were characterized by a bimodal pattern with a dominant peak in April, most likely due to anthropogenic Arctic haze, and a lower peak in July, most likely due to natural sources (e.g., in-cloud processing or biomass burning). In addition to oxalate, the highest abundance of sulfate (Fig. S5c) in F3 further suggests that the potential importance of in-cloud processing as a pathway leading to secondary aerosol.[10] The high abundance of NO_3^-, oxalate and K^+ in F3 may also indicate potential sources of aged biomass burning.[11] The days (10 – 17 July 2015) with highest concentrations of F3 also showed peaks in oxalate (**Figs. S7c and S8e**). This may be related to long-range transport of biomass burning aerosol.[12] Thus, F3 is identified as secondary aerosol, likely associated with anthropogenic sources in spring and natural sources in summer.

F4: High contributions to Zn (97.6%), Ni (95.8%), Cu (82.9%) and Cr (55%) (**Fig. S5d**). V and Ni are often used as tracers for shipping emissions, but the F4 is unlikely to be of this origin due to lower ratio of V/Ni (0.06, w/w) compared to that from shipping emissions (i.e., 3 – 5, w/w).[13] Besides, F4 is unlikely associated with industrial pollution during Arctic haze period, i.e., March

and April (**Fig. S7d**). Zn, Fe, Ni, Cu and Cr are the most abundant metal species in the F4 profile (**Fig. S5d**). The ratio of Ca^{2+}/Na^{+} and Mg^{2+}/Na^{+} for F4 are 0.038 and 0.1 (w/w), respectively, which are similar to ratios of 0.038 and 0.129 in seawater.[14] In addition, the PVSD in the coarse mode is similar to that from sea spray-related sources.[9] However, there is no enrichment of Cl^- in F4. Because of no clear source signature of this factor, F4 is labelled as unidentified trace metals in the present study. Note that this factor was found even using solutions of 6 – 8 factors (**Fig. S2**).

F5: High contributions (93.5%) to non-sea salt fraction of Na^+ (nss-Na^+), Ca^{2+} (nss-Ca^{2+}) and mineral fraction of SO_4^{2-} (mineral-SO_4^{2-}). The nss-Ca^{2+} shows a good agreement with Al (a tracer for mineral dust) variability (**Fig. S8f**), with R^2 of 0.69, indicating that nss-Ca^{2+} is a conservative tracer for mineral dust.[9] The ratios (w/w) of Ti/Al and Mn/Al are respectively 0.047 and 0.013, which are close to 0.047 and 0.009 from upper continental crust.[15] Thus, F5 is identified as mineral dust_1.

F6: High contributions to Pb (89.9%), Cd (88.6%), As (82.7%), anthropogenic fraction of SO_4^{2-} (anthr- SO_4^{2-}, 81.7%), Br^- (47.3%) and NH_4^+ (44.7%). Enhanced As, Cd and Pb are found during Arctic haze period.[16] The PNSD for F6 is similar to monthly average PNSD in March and April, characterized by a unimodal distribution at accumulation mode of ~200 nm. F6 dominated accumulation-mode particles with diameters of 171.5 nm – 0.835 μm (**Fig. 2**). Accumulation-mode particles were generally considered to be related to anthropogenic Arctic haze with largest enhancement in early spring.[9,17,18] The high concentrations of F6 in March and April (**Fig. S7f**) and the large contributions to Pb, Cd and As further supported that this factor is related to anthropogenic origins. F6 made a small contribution to oxalate, which might be originated from fossil fuel combustion.[19]

F7: High contributions to sea salt-related species, including sea-salt fraction of Na^+ (ss-Na^+, 92.0%) and Ca^{2+} (ss-SO_4^{2-}, 92.0%), Cl^- (89.6%), Mg^{2+} (77.2%) and K^+ (60.6%). The PVSD shows a unimodal distribution at a peak of 3 – 4 μm, which is similar to that of sea spray-related aerosol.[9] F6 is the largest contributor to coarse-mode particles with diameters of 0.835 – 6.264 μm (**Fig. 2**) and to PM_{10} (47.6%) among the nine factors. F7 is identified as sea salt.

F8: High contributions to Al (63.7%), Ba (82.6%), Ce (92.8%), Fe (67.7%), La (96.0%), Mn (65.3%), Ti (78.0%) and V (61.7%). The most abundant metal species in F8 are Al, Fe, Ti and Mn (**Fig. S5h**). The ratios (w/w) of Ti/Al and Mn/Al are respectively 0.055 and 0.015, which are close to 0.047 and 0.009 from upper continental crust.[15] Thus, F7 is identified as mineral dust_2. A similar temporal pattern (**Figs. S7e and h**) and a good correlation (R^2=0.46, **Fig. S4**) between F5 and F8 suggest that they are likely from a common source.

F9: High contributions to Br^- (33%). F9 dominated particles with diameters of 6.264 – 19.81 μm (**Fig. 2**). The most abundant ion species are sea salt-related species, such as Cl^-, ss-Na^+, ss-SO_4^{2-} and Mg^{2+} (**Fig. S5i**). For metal elements, they are mineral dust-related species, such as Al, Fe and Ti. Br^- is the major species that distinguished F9 from sea salt/mineral dust despite a weak correlation (R^2=0.26, **Fig. S4**) between F9 and sea salt factor. The Br^-/Na enrichment (calculated by $([Br]/[Na])_{obs}/([Br]/[Na])_{seawater}$, the $([Br]/[Na])_{seawater}$ mass ratio[14] is 0.006) for F9 is 12.05, suggesting it is less likely to originate from sea spray-related sources. The high Br^-/Na

enrichment is likely related to blowing snow, because: (i) snowfall could increase the Br^-/Na enrichment in surface snow;[20] (ii) high concentrations of F9 were often accompanied by high wind speed, boundary layer height and snowfall (**Fig. S10**); and (iii) the PVSD is dominated by coarse mode, with a peak diameter of 3 - 4 µm, which is slightly larger than the sea salt factor. F9 was the largest contributor to particles larger than 6.264 µm diameter. Note that the ratios (w/w) of Ti/Al and Mn/Al are 0.063 and 0.008, respectively, are close to those from upper continental crust.[15] This suggests that F9 presents a fraction of mineral dust despite mineral dust not being abundant. Thus, F9 is identified as blowing snow, with some contribution from mineral dust and sea salt. However, it is possible that observed aerosol produced from blowing snow in real world is a combination of F8 and F9 from a perspective of factor analysis.

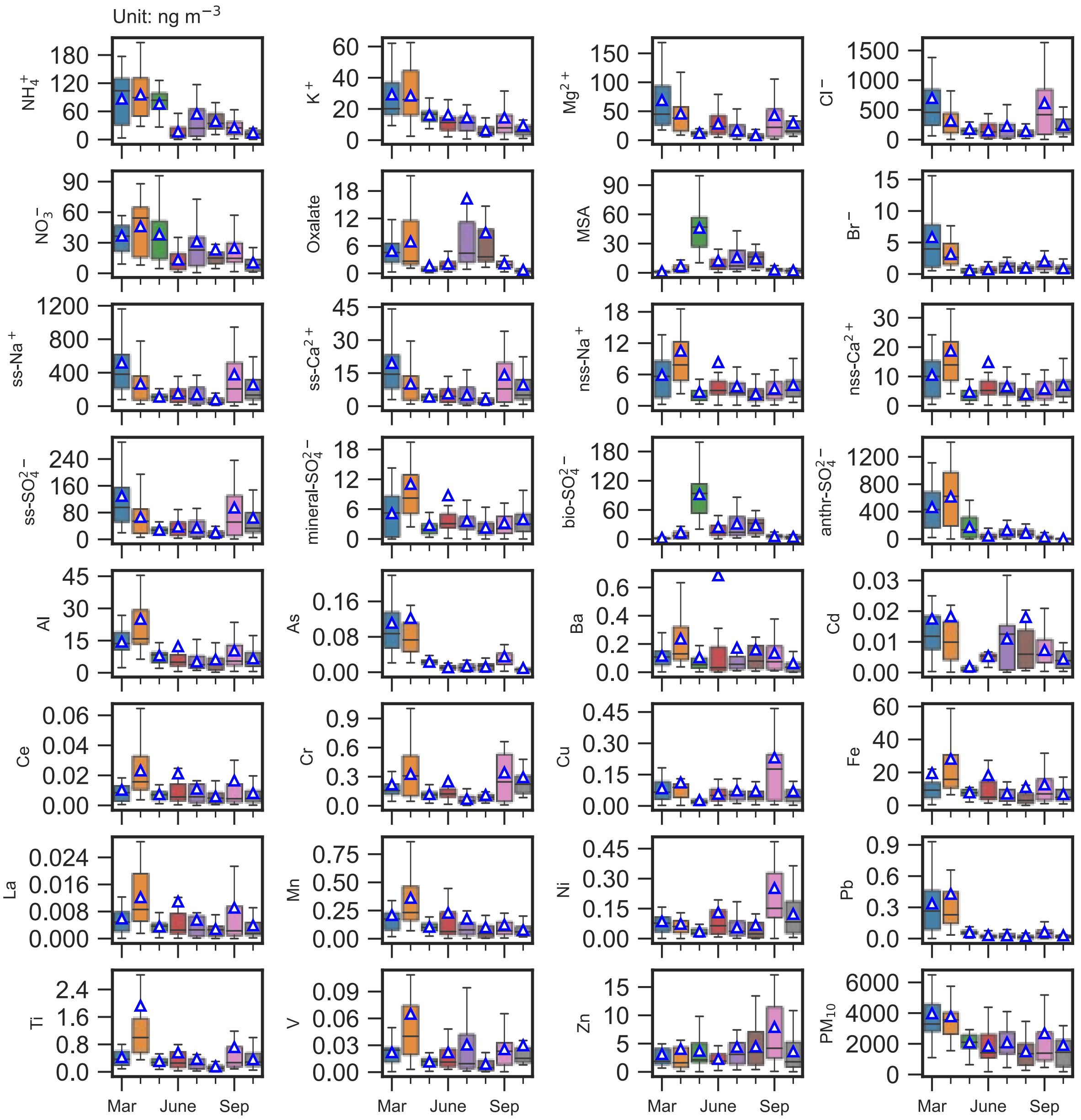

Fig. S1. Box plots of mass concentration (unit: ng m^{-3}) of the chemical species in each month. Box plots display the median (center line), interquartile range (box) and 1.5 times the interquartile range (whiskers). The mean values are marked as triangles.

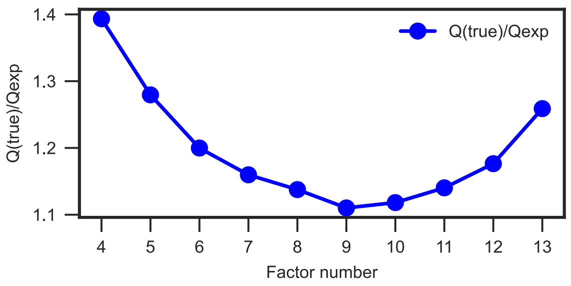

Fig. S2a. The ratio between Q(true) and Qexp decreased from 4-factor solution to 9-factor solution, and then increased from 9-factor solution to 13-factor solution.

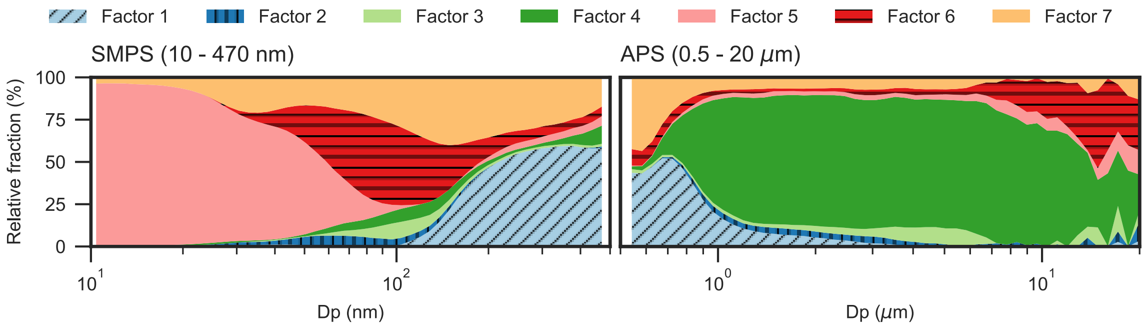

Fig. S2b. Size-resolved factor contributions for a 7-factor solution.

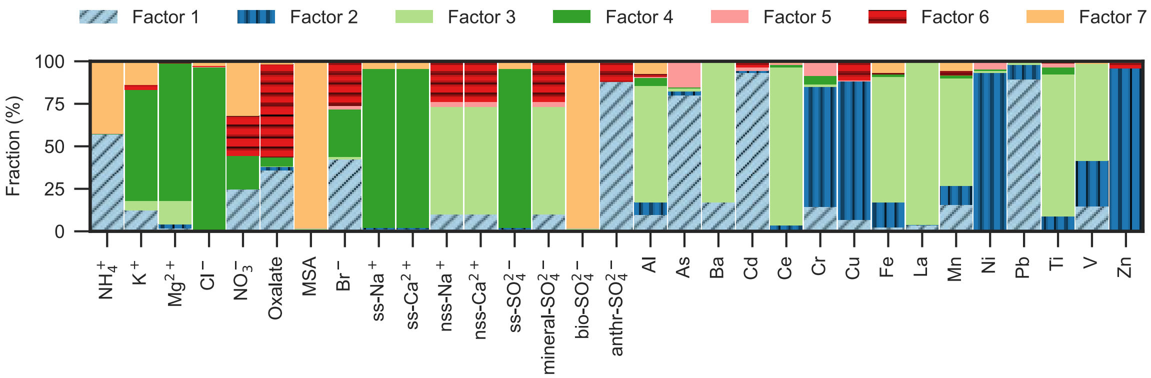

Fig. S2c. Relative contributions for each chemical species for a 7-factor solution. A 7-factor solution resulted in the following: Factor 1 - anthropogenic, Factor 2 - trace metals, Factor 3 - mineral dust, Factor 4 - sea salt, Factor 5 - nucleation, Factor 6 - secondary, Factor 7 - biogenic.

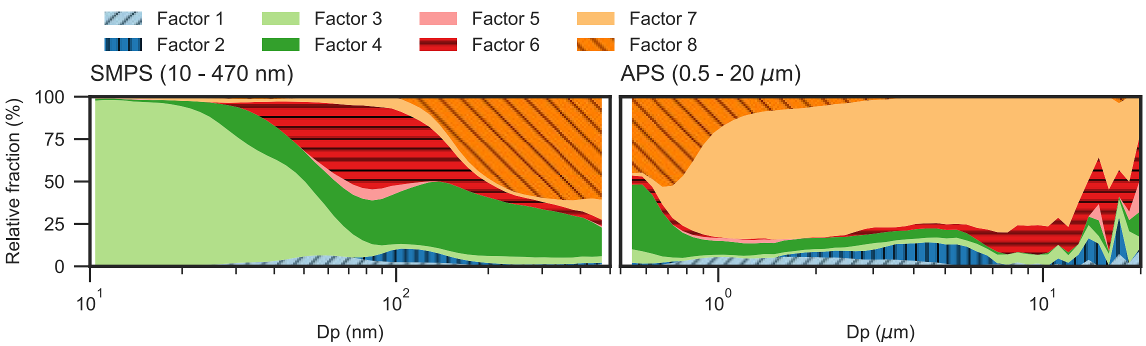

Fig. S2d. Size-resolved factor contributions for an 8-factor solution

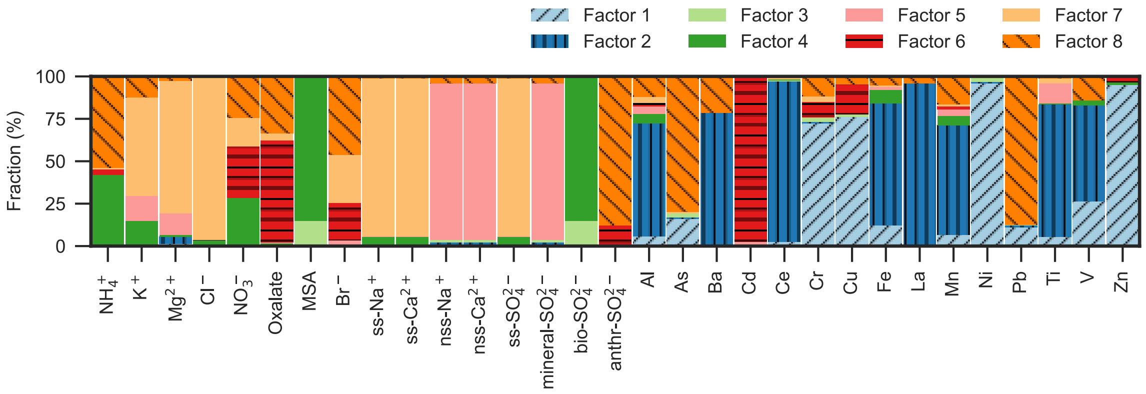

Fig. S2e. Relative contributions for each chemical species for an 8-factor solution. An 8-factor solution resulted in the following: Factor 1 - trace metals, Factor 2 - mineral dust_1, Factor 3 - nucleation, Factor 4 - biogenic, Factor 5 - mineral dust_2, Factor 6 - sea salt, Factor 7 - anthropogenic.

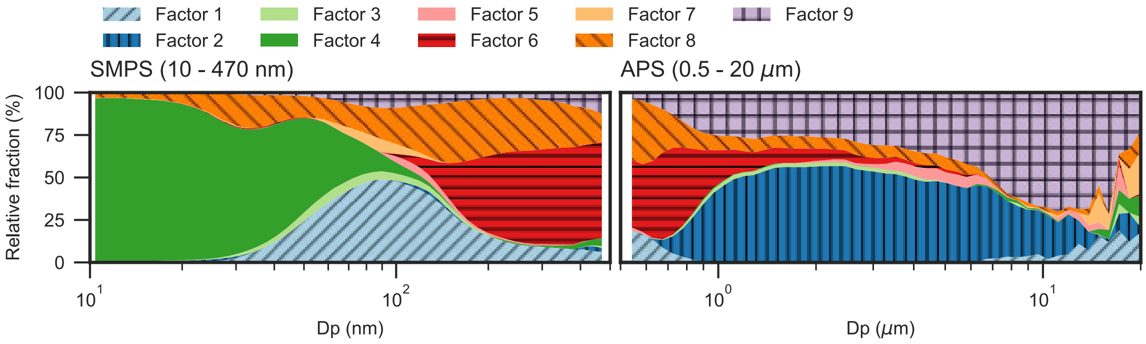

Fig. S2f. Size-resolved factor contributions for a 9-factor solution

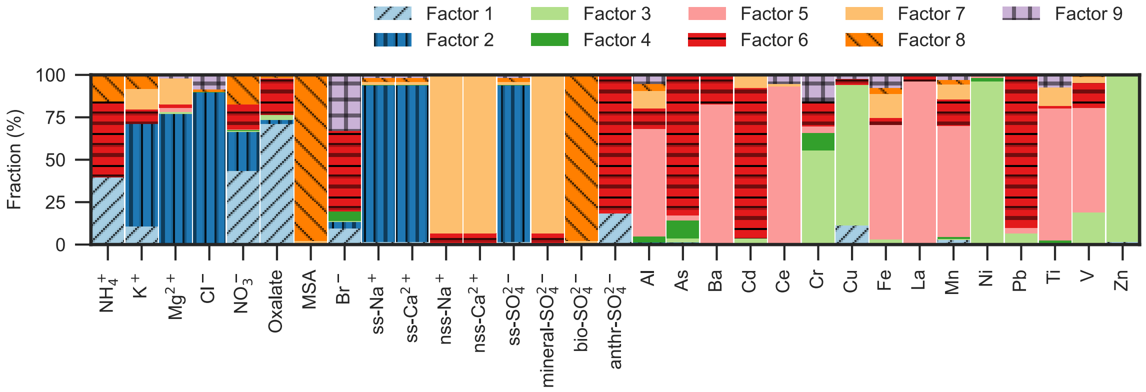

Fig. S2g. Relative contributions for each chemical species for a 9-factor solution. A 9-factor solution resulted in the following: Factor 1 - secondary, Factor 2 - sea salt, Factor 3 - trace metals, Factor 4 - nucleation, Factor 5 - mineral dust_1, Factor 6 - anthropogenic, Factor 7 - mineral dust_2, Factor 8 - biogenic, Factor 9 - blowing snow.

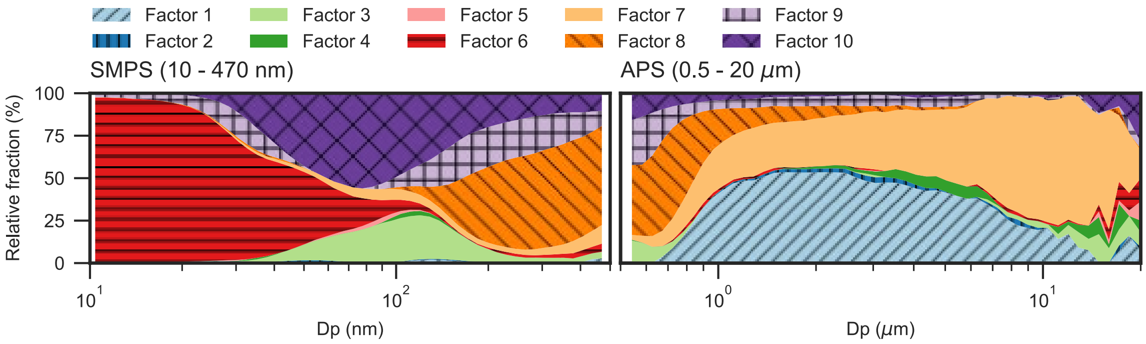

Fig. S2h. Size-resolved factor contributions for a 10-factor solution.

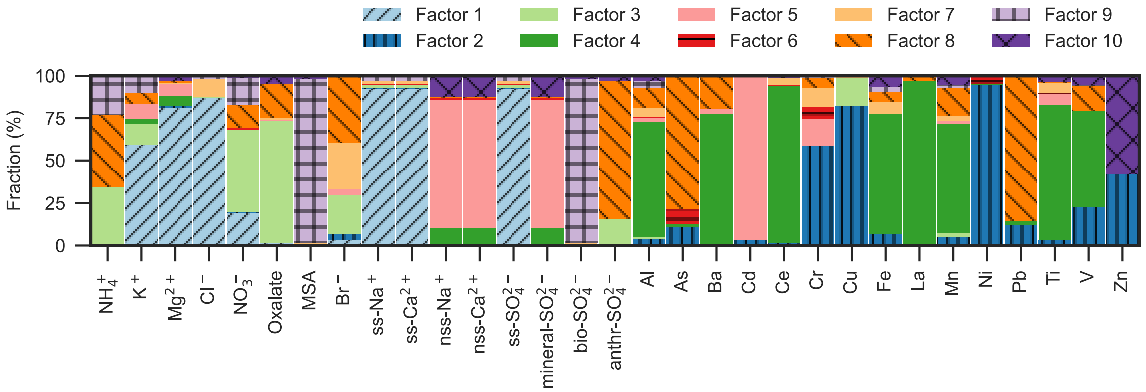

Fig. S2i. Relative contributions for each chemical species for a 10-factor solution. A 10-factor solution gave factors of Factor 1: sea salt, Factor 2: trace metals, Factor 3: secondary, Factor 4: mineral dust_1, Factor 5: mineral dust_2, Factor 6: nucleation, Factor 7: blowing snow, Factor 8: anthropogenic, Factor 9: biogenic, Factor 10: Zinc-related.

Fig. S2. Particle size distribution profiles, chemical profiles and Q(true)/Qexp for the solutions with different factor number. The ratio of Q(true)/Qexp for a 9-factor solution is closest to 1 (**Fig. S1a**), indicating a 9-factor solution might be the optimal solution. **Figs. S1b-i** show particle size distribution (PSD) profiles and chemical profiles for factor solutions from 7-factor solution to 10-factor solution. When factor number increases from 8 to 9, a blowing snow factor is separated from sea salt factor. A blowing snow factor is expected as there are two type of sea-salt aerosol cluster found in Svalbard according to a previous study.[9] When factor number increases from 9 to 10, a zinc-related factor is separated from the trace metal factor from 9-factor solution. This additional factor doesn't relate to any other chemical species, indicating this factor might be artifactual from PMF modelling. Thus, a 9-factor solution was chosen as the optimal solution.

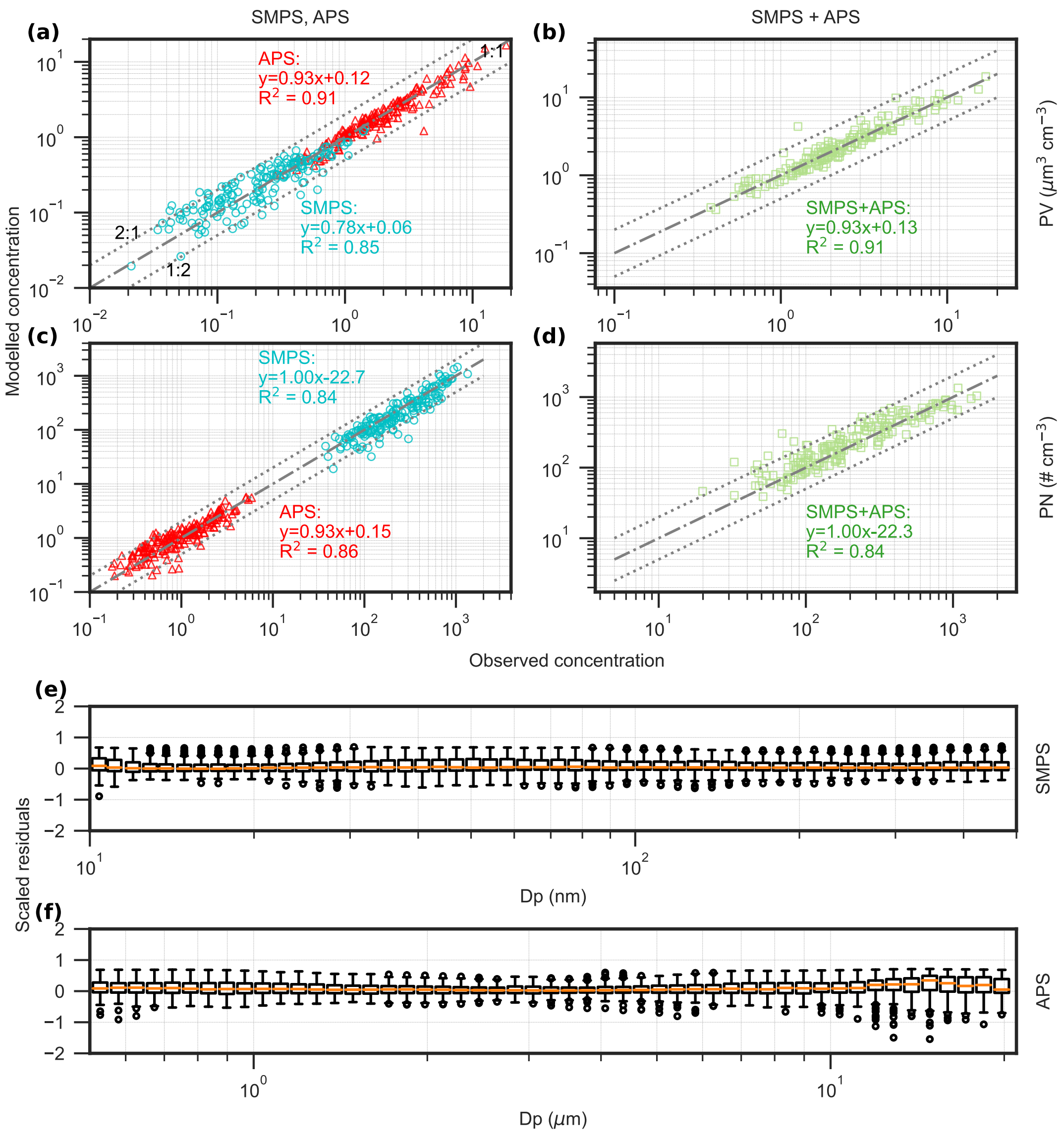

Fig. S3. Correlations between modelled and observed concentration for particle volume and number concentrations. Correlations between modelled and observed concentration for particle volume concentration from diameters covered by **(a)** SMPS (i.e., 10 - 470nm), APS (0.5 - 20 µm) and **(b)** combined SMPS and APS (10 nm - 20 µm). Correlations between modelled and observed concentration for particle number concentration from diameters covered by **(c)** SMPS (i.e., 10 - 470 nm), APS (0.5 - 20 µm) and **(d)** combined SMPS and APS (10 nm - 20 µm). Box plot of scaled residues from the nine-factor solution for particles with diameters measured by **(e)** SMPS and **(f)** APS. The scaled residuals within the ranges of ±3 are generally considered as normal distribution. Box plots display the median (centre line), interquartile range (box) and 1.5 times the interquartile range (whiskers). The data points exceed the whiskers are noted as black circles.

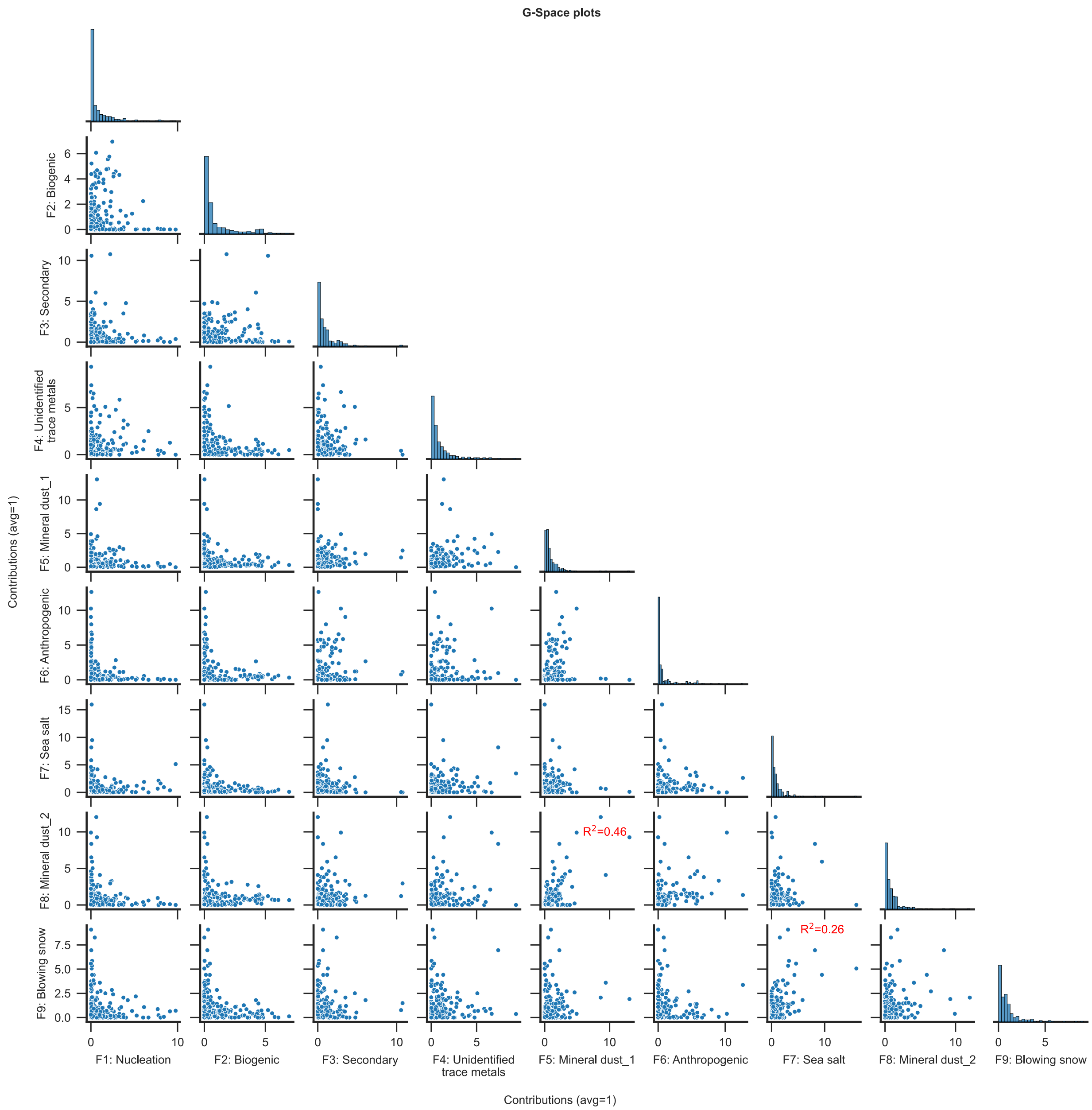

Fig. S4. Paired scatter plot between two aerosol factors. The figures show that most of paired factors are well separated, however, weak correlations were found for mineral dust_1 versus mineral dust_2, sea salt versus blowing snow.

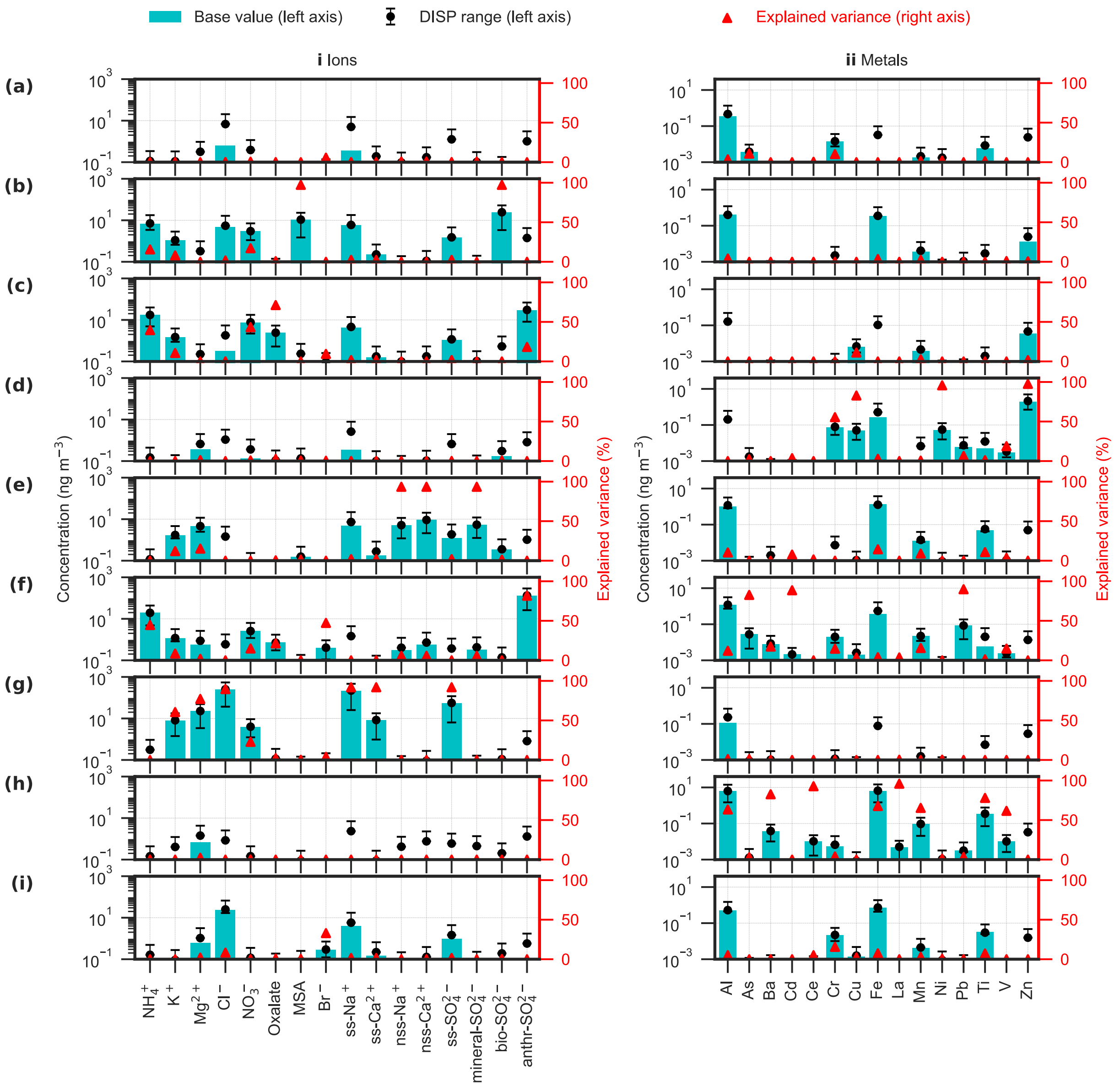

Fig. S5. Chemical profile of the nine factors resolved by PMF model. The nine factors are: **(a)** F1-nucleation aerosol, **(b)** F2 – biogenic aerosol, **(c)** F3 – secondary aerosol, **(d)** F4 – unidentified trace metals, **(e)** F5 – mineral dust_1, **(f)** F6 – anthropogenic aerosol, **(g)** F7 – Sea salt, **(h)** F8 – mineral dust_2 and **(i)** F9 – blowing snow. The left and right panels are chemical profiles for ions and trace metals, respectively. The bars are the base values, the asymmetric error bar represent the maximum and minimum displacement (DISP) values (left y-axis). The red triangle represents the % explained variance (right y-axis). Logscale is applied to left y-axis of each subfigure. Details about source identification are discussed in **Supplementary section 2**.

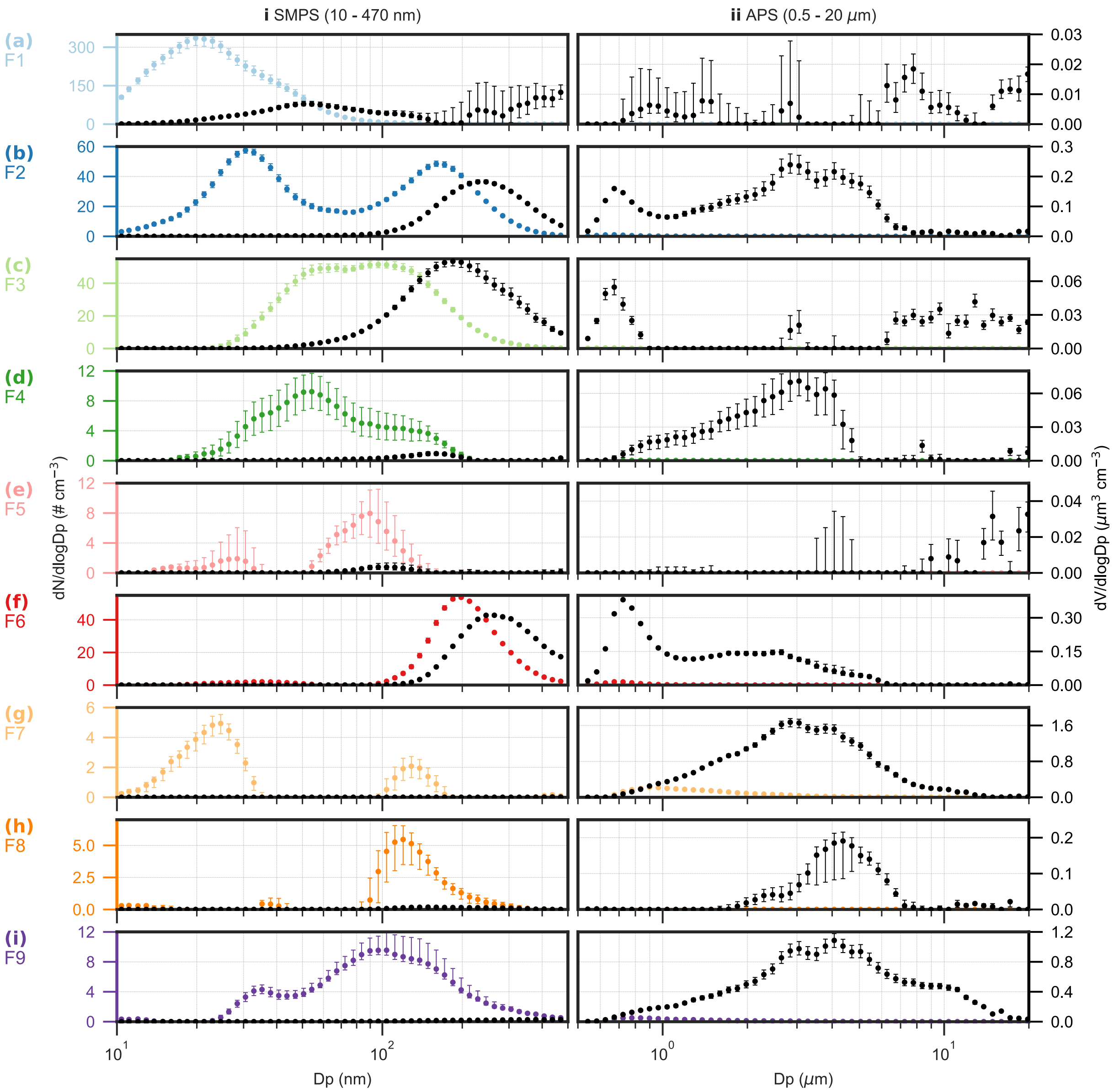

Fig. S6. Particle size distribution profiles for the nine resolved factors. The nine aerosol factors from top to bottom are: **(a)** F1 nucleation factor, **(b)** F2 biogenic factor, **(c)** F3 secondary factor, **(d)** F4 unidentified trace metals, **(e)** F5 mineral dust_1, **(f)** F6 anthropogenic factor, **(g)** F7 sea salt, **(h)** F8 mineral dust_2 and **(i)** F9 blowing snow, respectively. The dot means the base value and the error bar means the displacement (DISP) range. Particle size distributions with black color are particle volume size distribution (right y-axis). Particle size distributions with other colors are particle number size distribution (left y-axis). Low DISP ranges for all size bins indicate the profiles are robust.

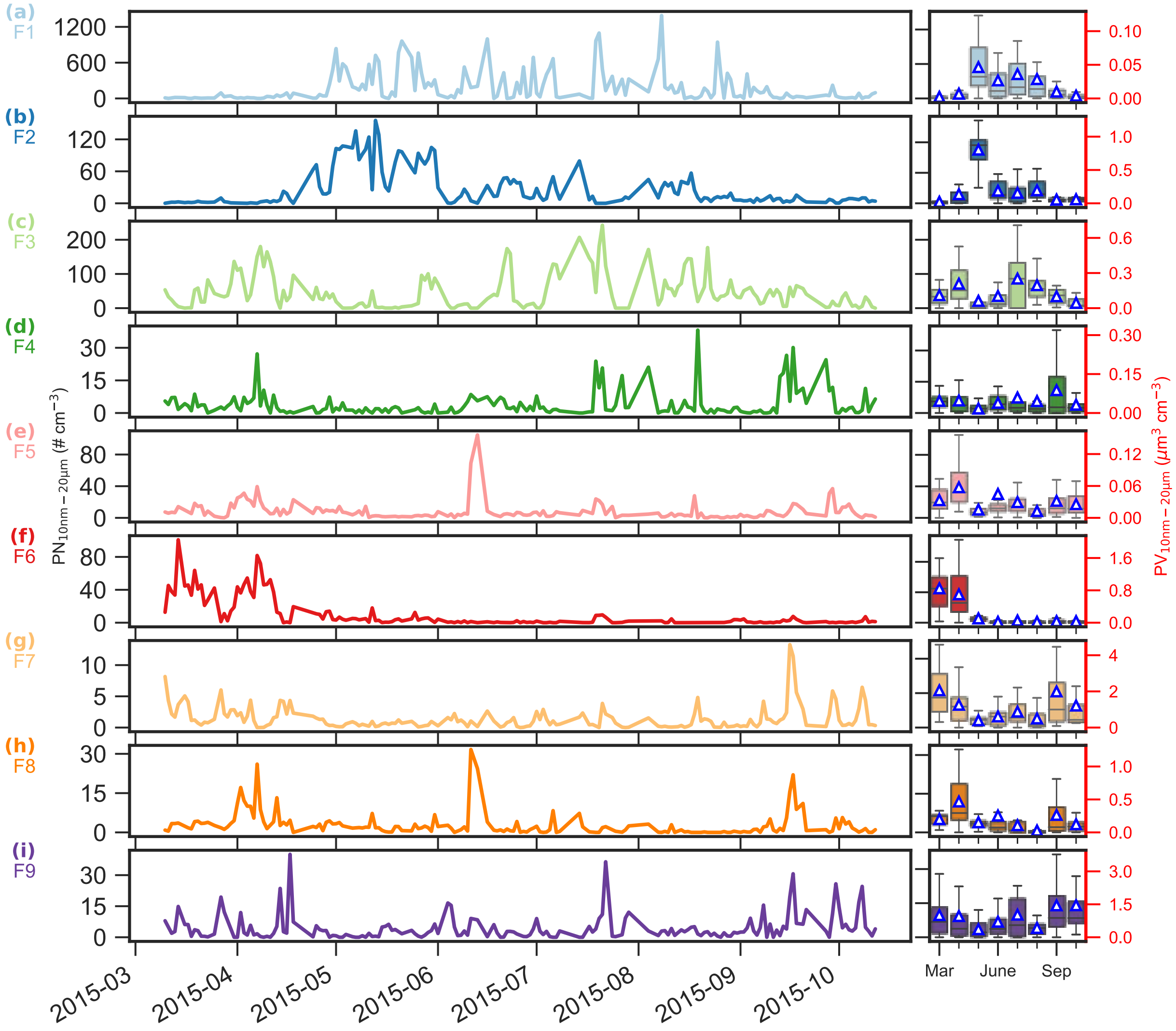

Fig. S7. Daily and monthly variation of particle number and volume concentrations for each aerosol factor. The nine factors from top to bottom are: **(a)** F1 nucleation factor, **(b)** F2 biogenic factor, **(c)** F3 secondary factor, **(d)** F4 unidentified trace metals, **(e)** F5 mineral dust_1, **(f)** F6 anthropogenic factor, **(g)** F7 sea salt, **(h)** F8 mineral dust_2 and **(i)** F9 blowing snow, respectively. The left panel shows daily concentrations for each aerosol factor. The right panel shows box plot of each aerosol factor in each month. Box plots display the median (centre line), interquartile range (box) and 1.5 times the interquartile range (whiskers). The mean values are marked as triangles. The left y-axis is for particle number concentration and the right y-axis is for particle volume concentration.

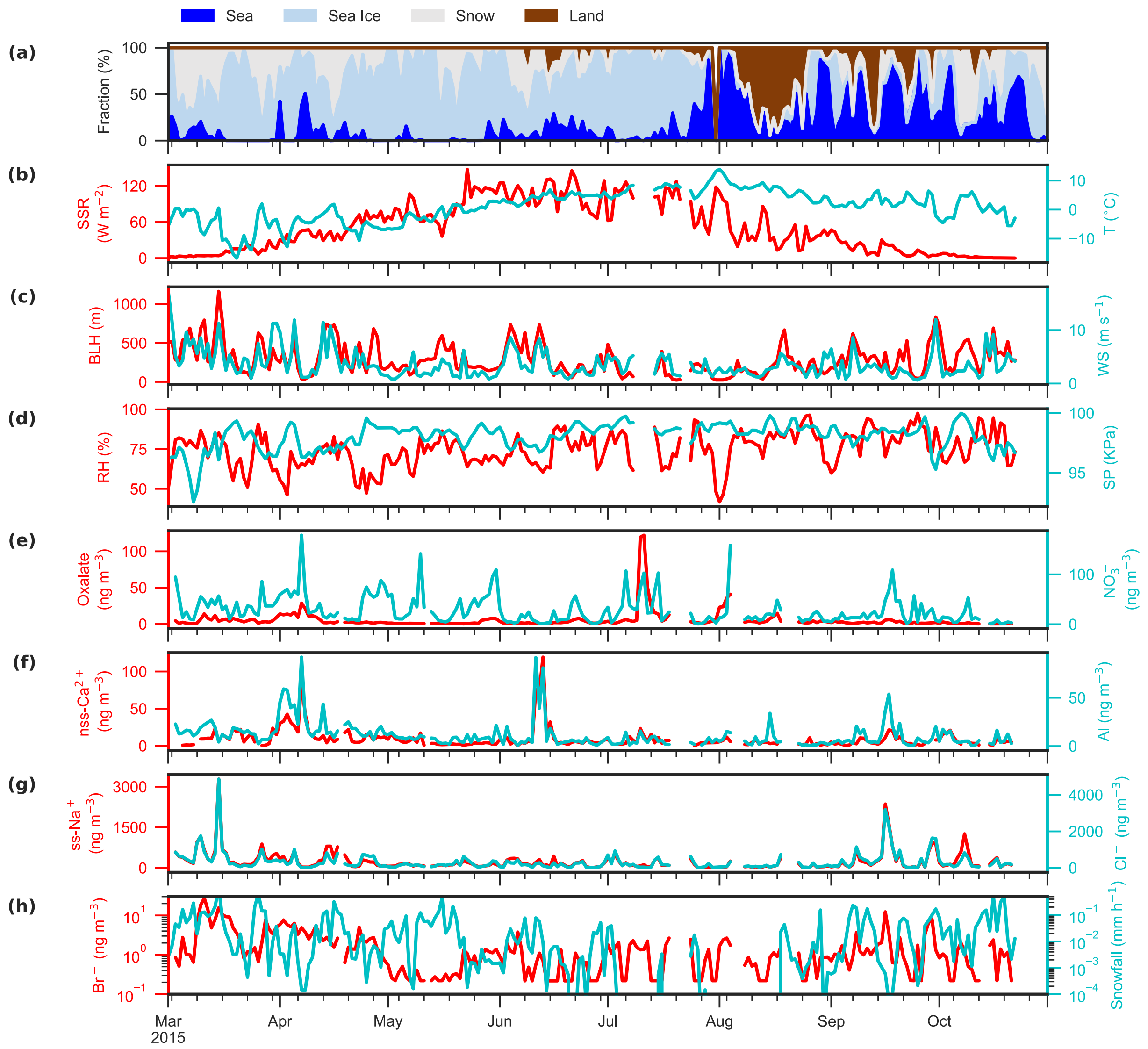

Fig. S8. Daily variations of different meteorological and chemical variables. The variables include **(a)** relative fraction of surface types that air masses have travelled over, **(b)** surface net solar radiation (SSR, left y-axis) and temperature (T, right y-axis), **(c)** boundary layer height (BLH, left y-axis) and wind speed (WS, right y-axis), **(d)** relative humidity (RH, left y-axis) and surface pressure (SP, right y-axis), **(e)** oxalate concentration (left y-axis) and NO_3^- concentration (right y-axis), **(f)** nss-Ca^{2+} concentration (left y-axis) and Al concentration (right y-axis), **(g)** ss-Na^+ (left y-axis) and Cl^- (right y-axis) concentration, **(h)** Br^- concentration (left y-axis) and snowfall (right y-axis).

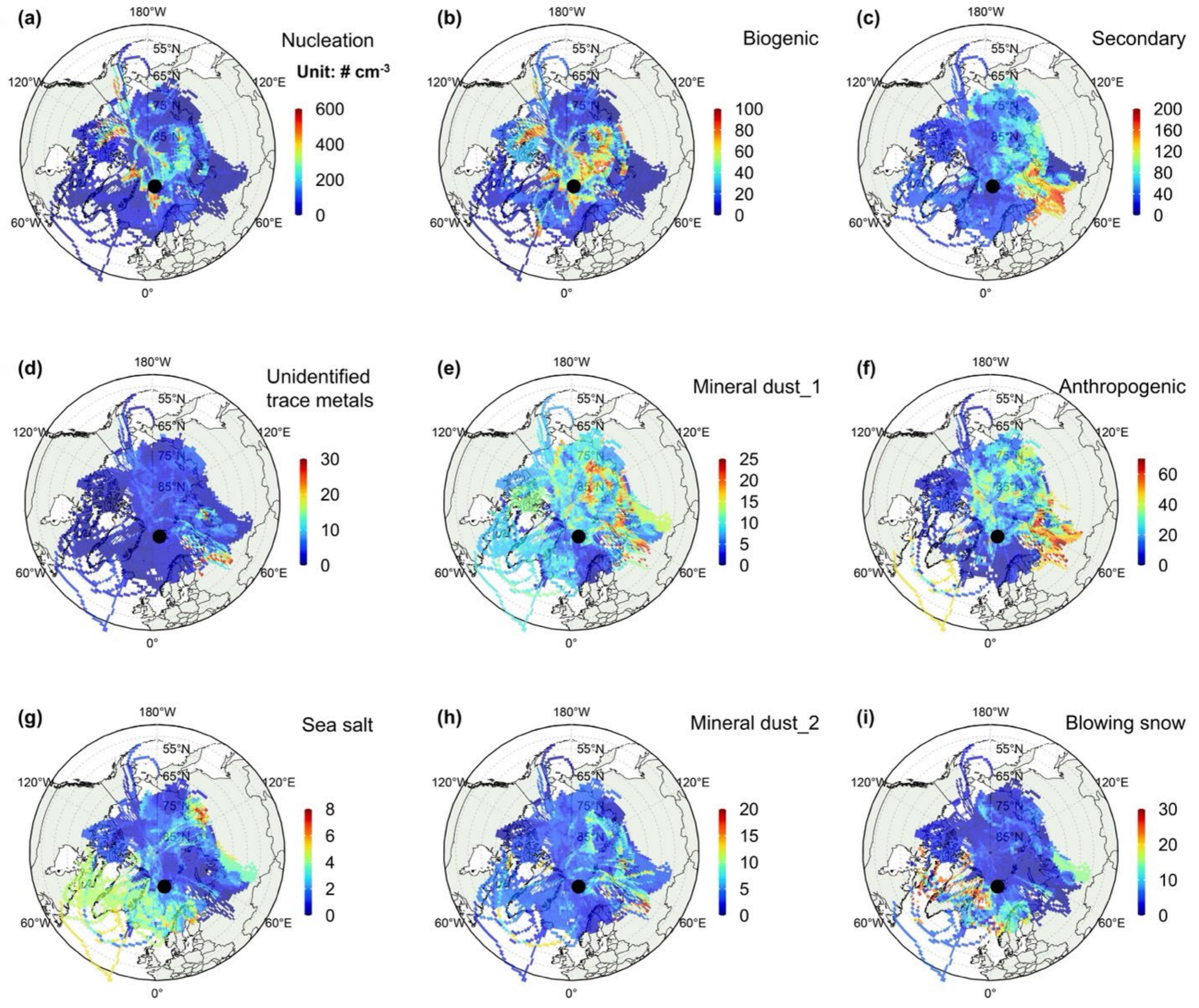

Fig. S9(a-i): Spring (i.e., March - May)

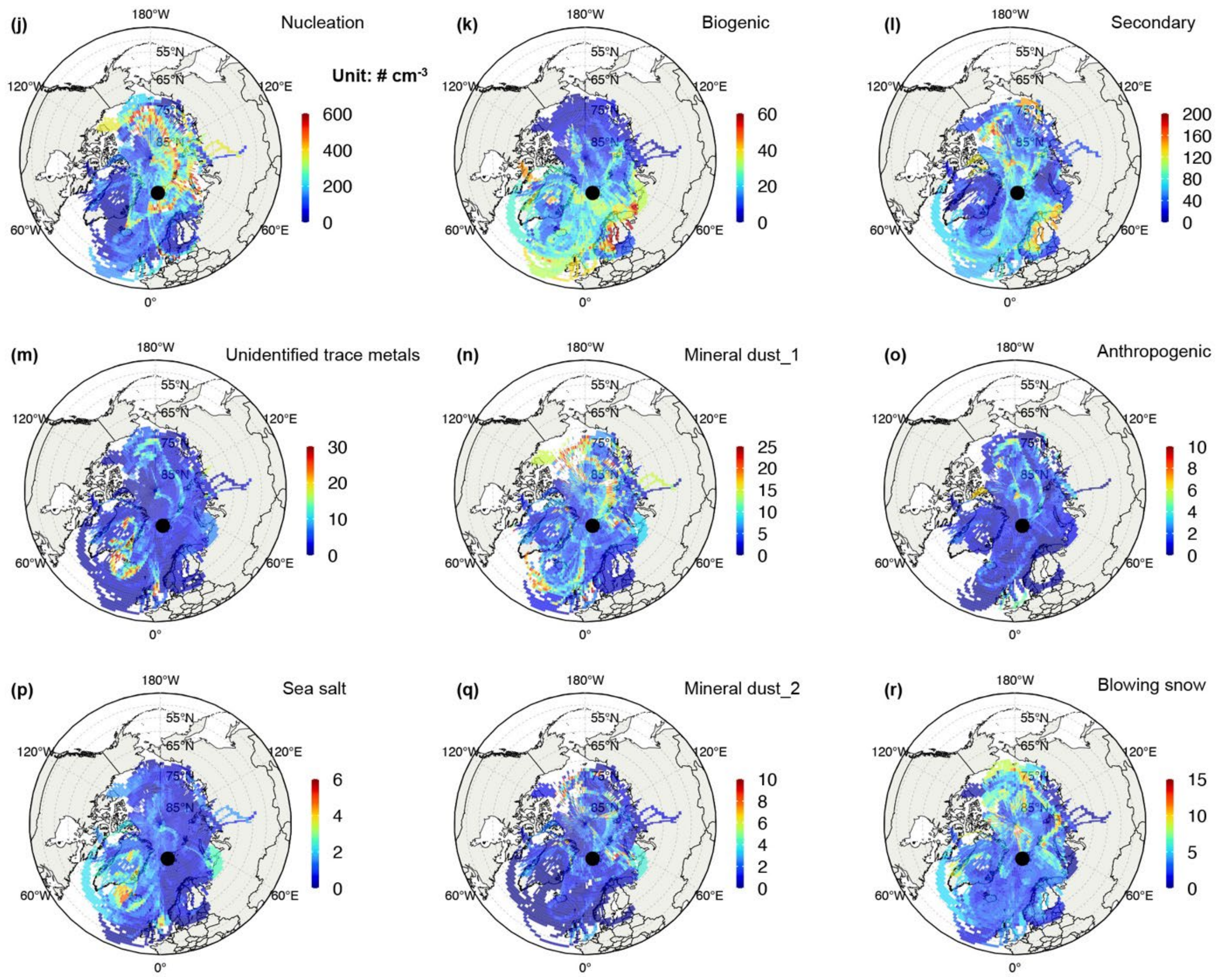

Fig. S9(j-r): Summer (i.e., June - August)

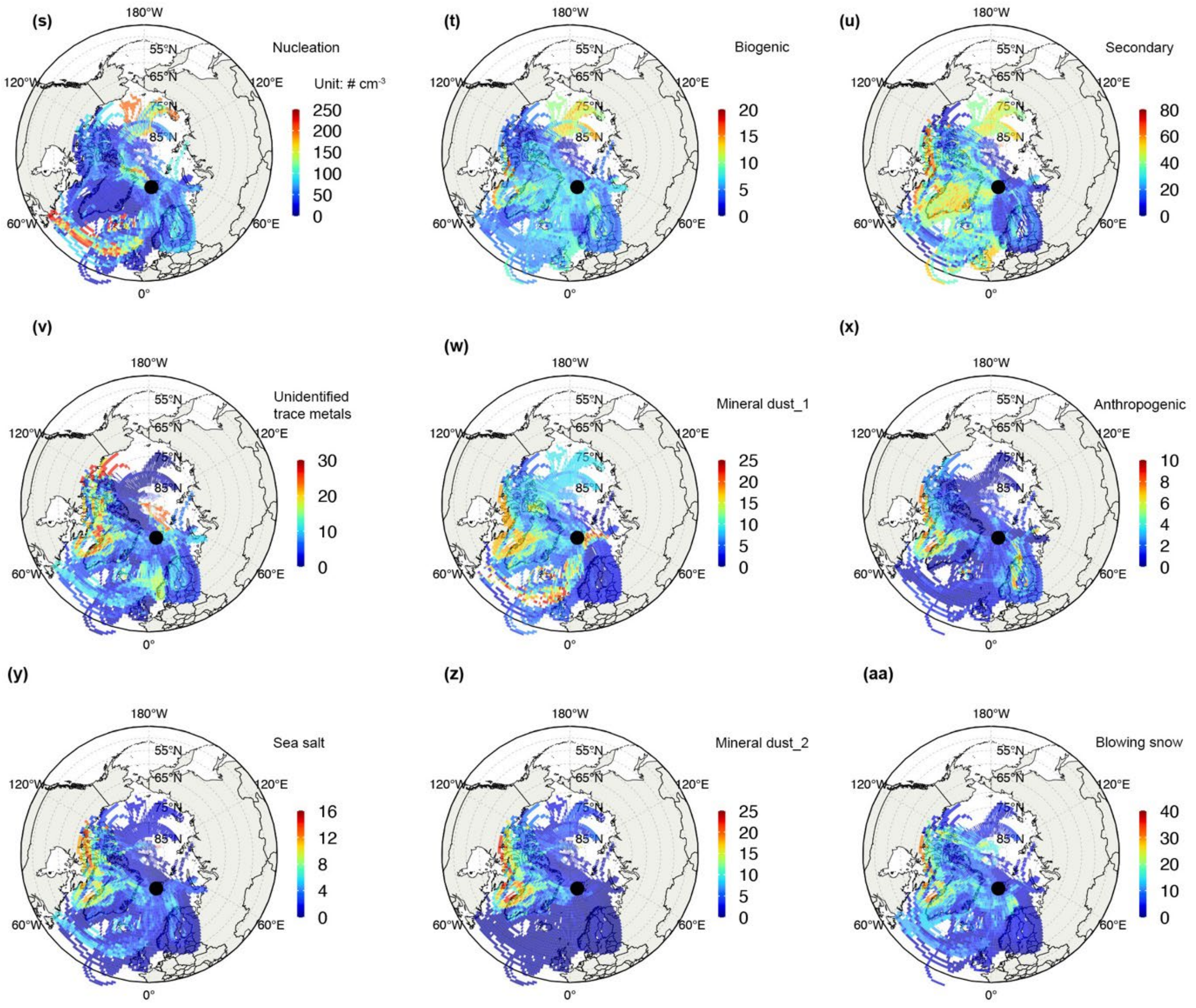

Fig. S9(s-aa): autumn (i.e., September - October)

Fig. S9. Maps of concentration weighted by trajectories (CWT) in spring (a-i), summer (j-r) and autumn (s-aa) for each aerosol factor.

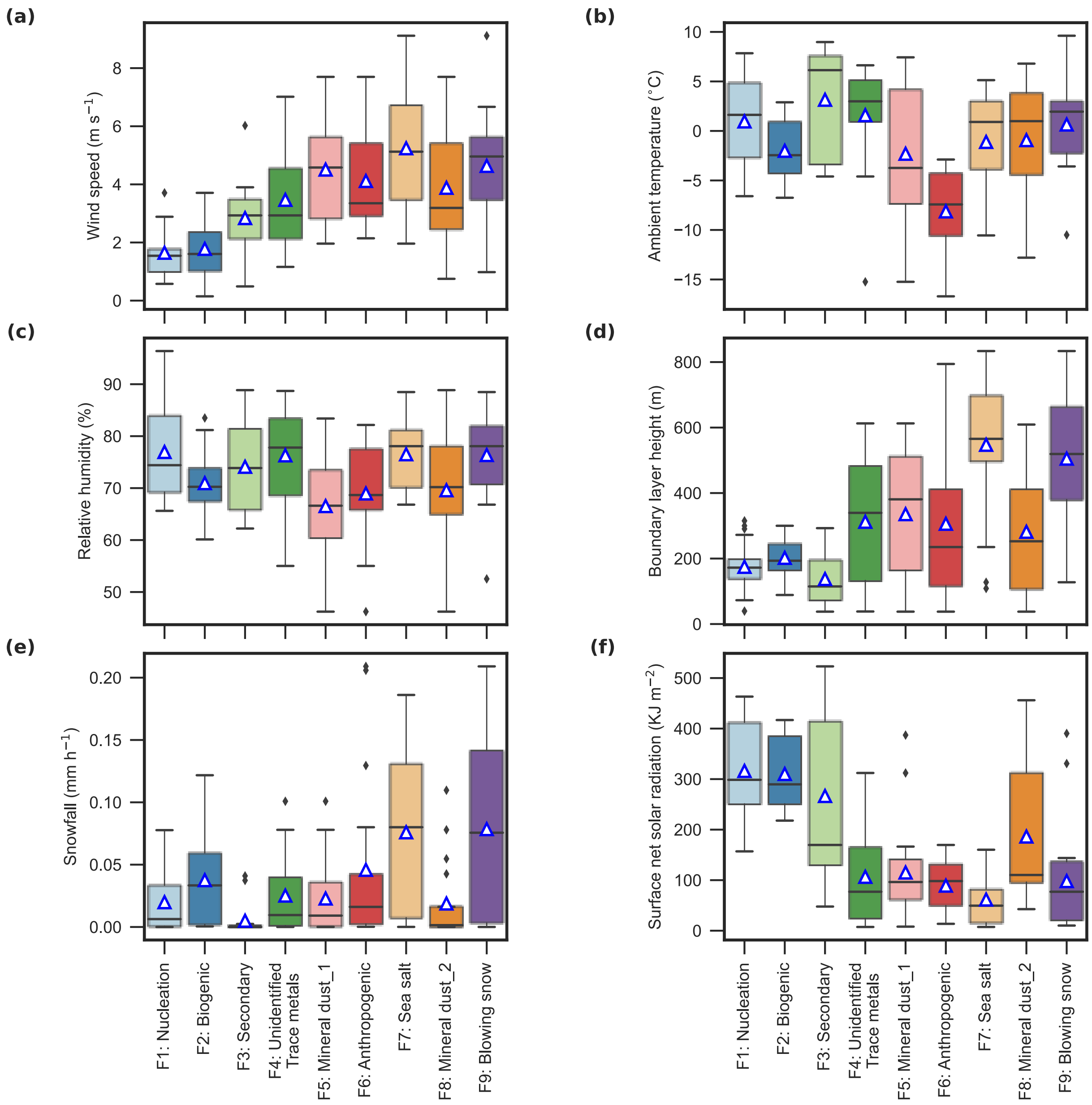

Fig. S10. Box plot of the meteorological parameters for aerosol concentrations that exceeded their 90th-percentile values within each factor. Box plots display the median (center line), interquartile range (box) and 1.5 times the interquartile range (whiskers). The mean values are marked as triangles. The data points exceed the whiskers are noted as black diamonds.

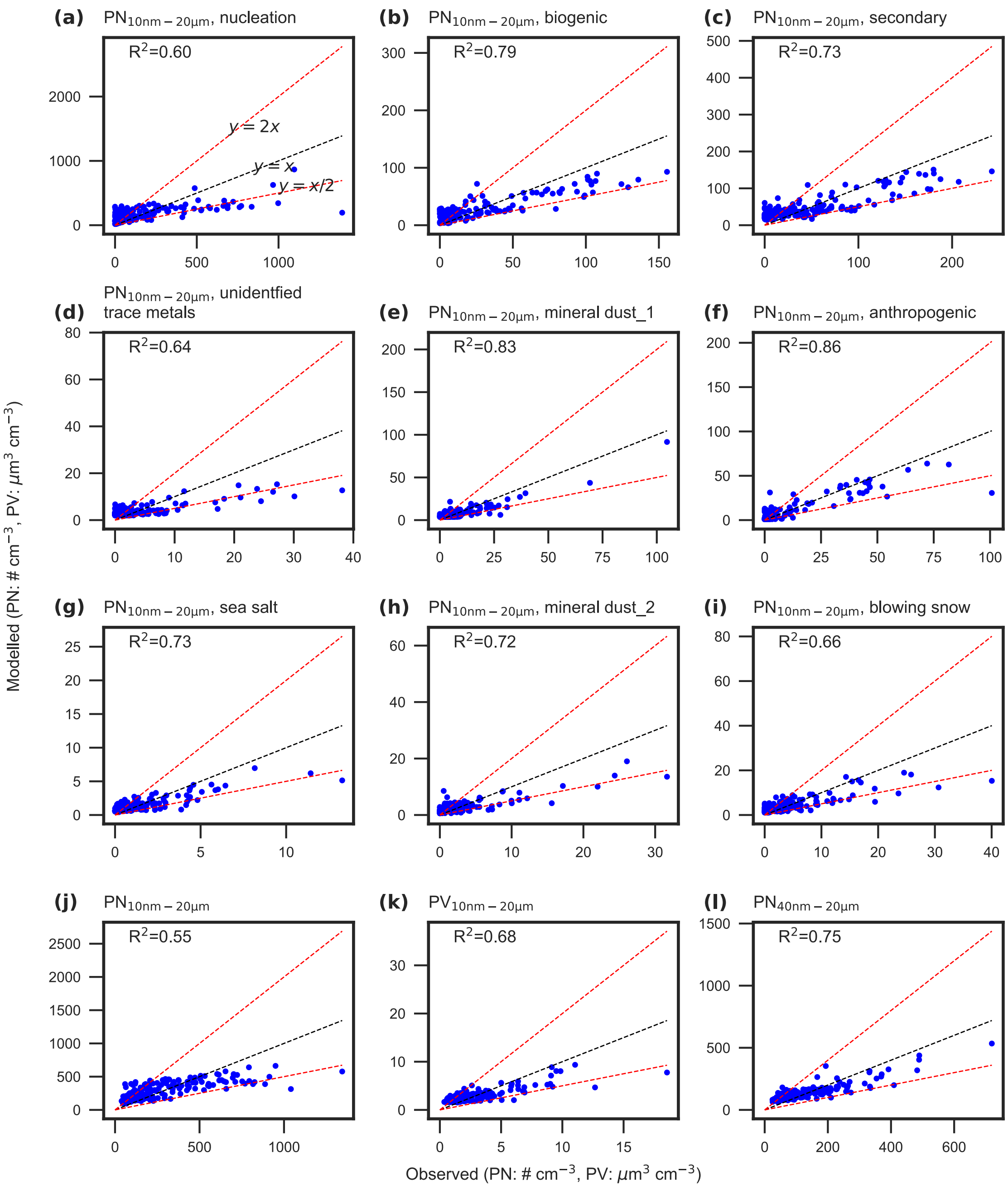

Fig. S11. Correlations between observed concentrations and modelled concentrations from the test data set of the random forest models. The test data set is one quarter from the whole dataset. The black-dash line is y=x and the two red-dash lines are y=2x and y=x/2, respectively.

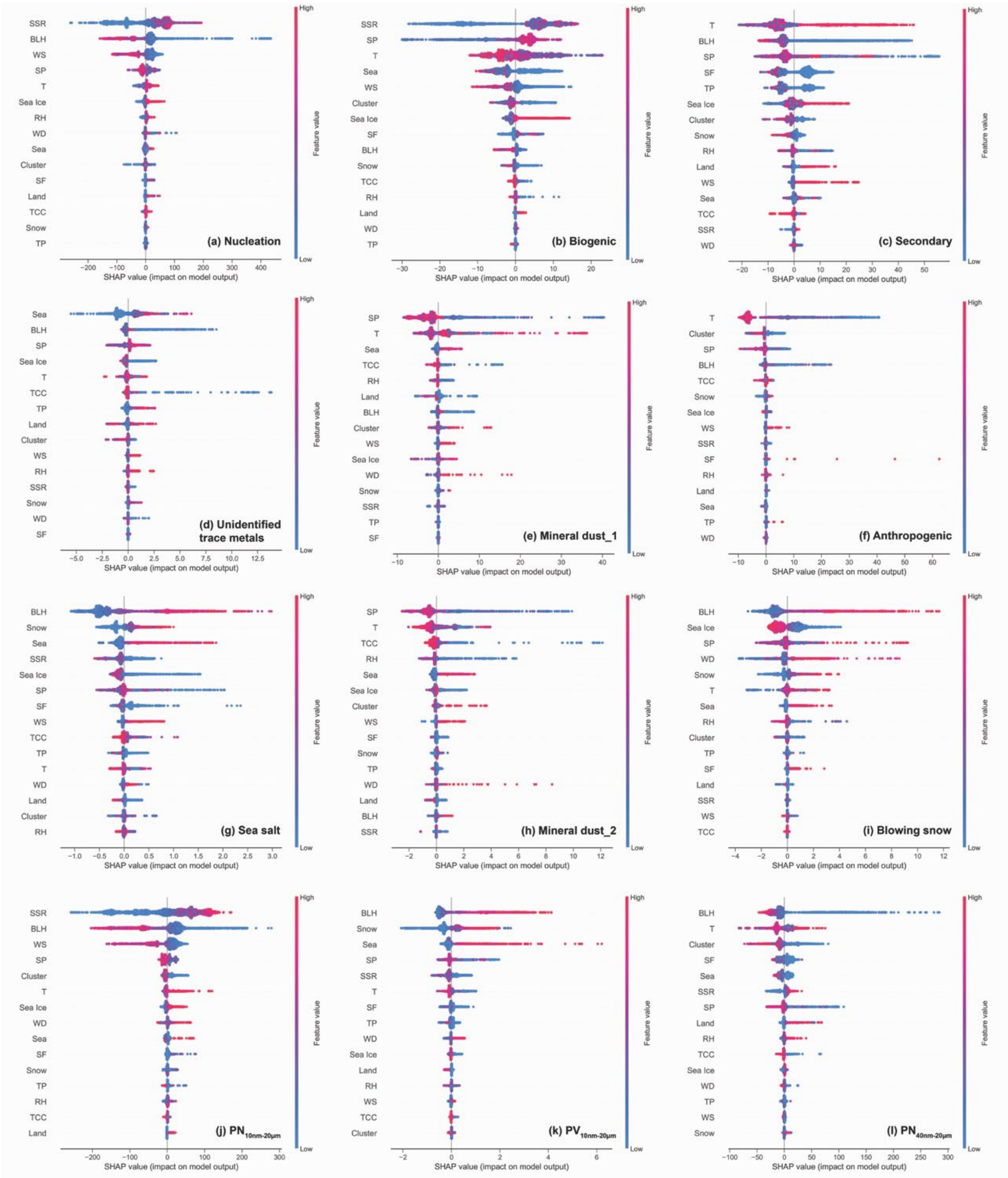

Fig. S12. Importance of different parameters and their impacts on concentrations of each aerosol source. The rank of the parameters (SSR: surface solar radiation, BLH: boundary layer height, WS: wind speed, SP: surface pressure, RH: relative humidity, WD: wind direction, Cluster: air mass cluster, SF: snowfall, TCC: total cloud cover, TP: total precipitation) from top to bottom for each subfigure represents the importance of the parameters to total SHAP values for each aerosol factor (see **Methods**), determined by ranking of |SHAP| of parameters. Take nucleation factor as an example, the six most important parameters are solar radiation, boundary layer height, wind speed, surface pressure, temperature and sea ice. Solar radiation, temperature and sea ice exposure have positive responses. Boundary layer height and wind speed have negative responses. Surface pressure has mixing effect on nucleation factor.

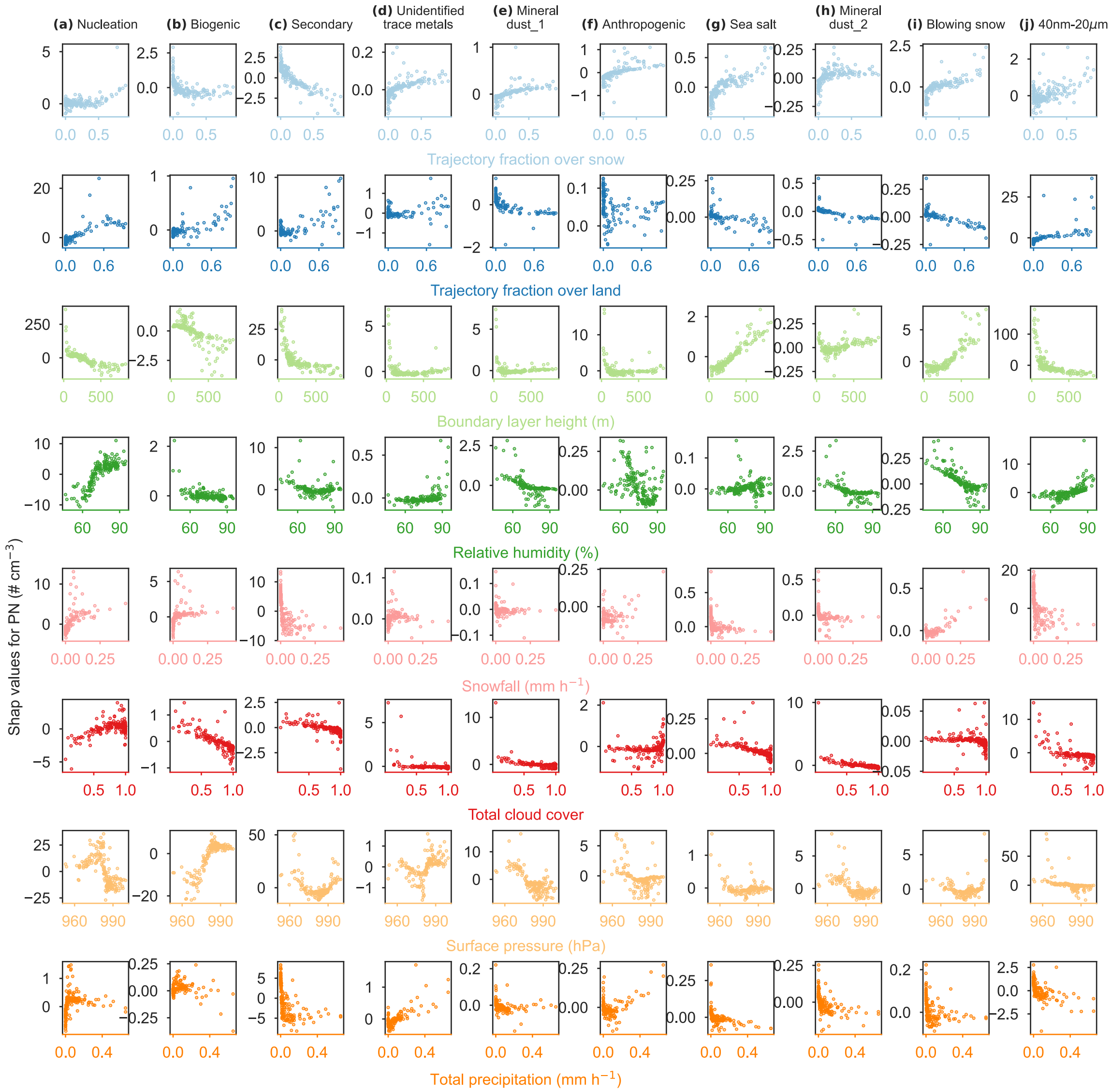

Fig. S13. SHAP dependence plot of each variable versus its SHAP value for each aerosol factor. This figure is similar to **Fig. 3** but shows SHAP dependence of additional variables not shown in **Fig. 3.**

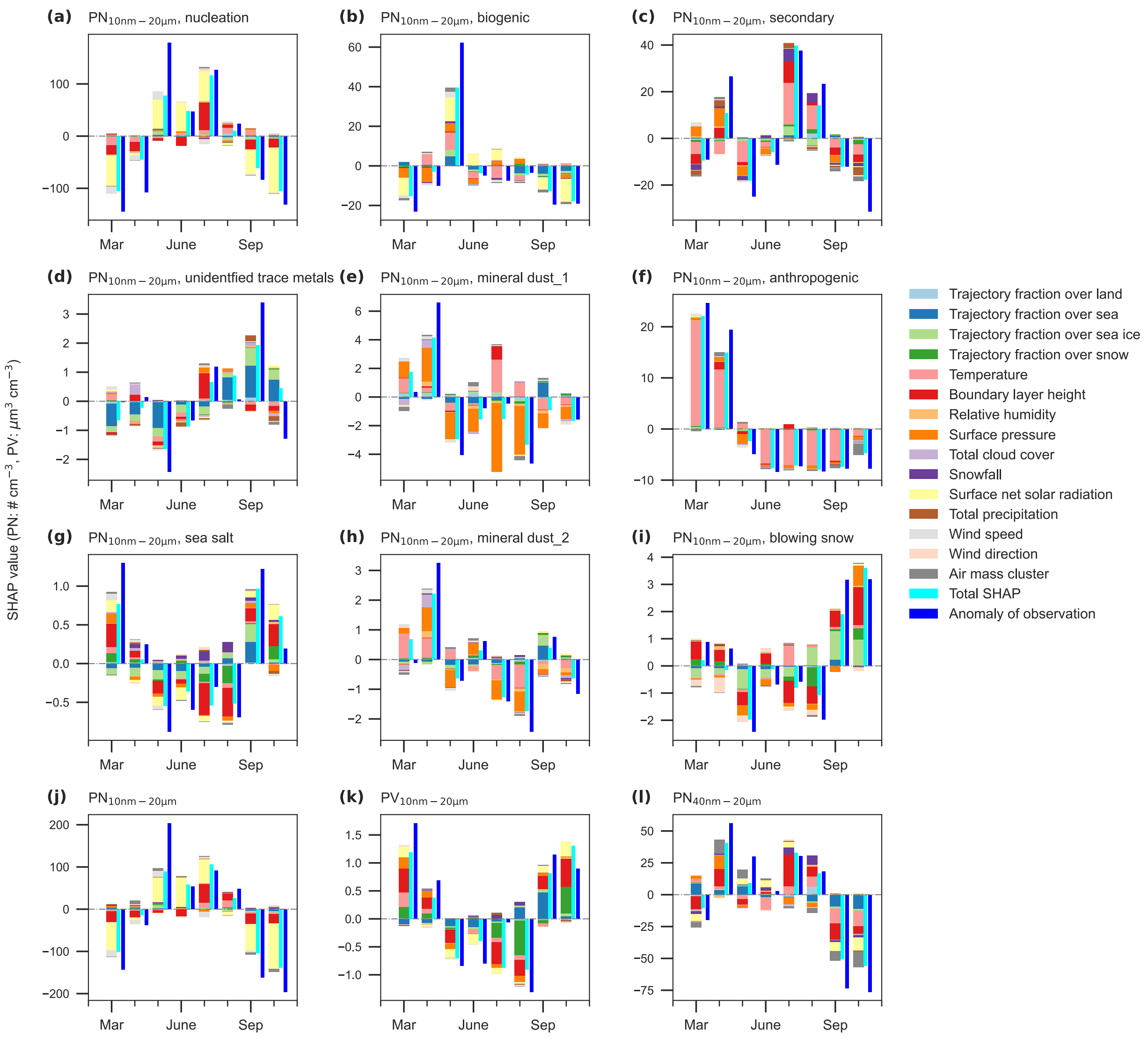

Fig. S14. Environmental drivers of monthly variation for the identified aerosol factors and total aerosol. The stacked bar plots show additive effects of positive responses (SHAP value above 0) as well as negative responses (SHAP value below 0) from the various parameters for **(a)** nucleation, **(b)** biogenic, **(c)** secondary, **(d)** unidentified trace metals, **(e)** mineral dust_1, **(f)** anthropogenic, **(g)** sea salt, **(h)** mineral dust_2, **(i)** blowing snow aerosols, total aerosol **(j)** in number and **(k)** in volume, and **(l)** particle number concentration at the diameter range of 40 nm - 20 µm. The anomaly of observation represents the anomaly of aerosol factor concentrations resolved by PMF. The total SHAP value and anomaly of concentration are denoted as bars besides each stacked bar. The trajectory fraction over land, sea, sea ice and snow represent relative fractions of the accumulated time for air masses travelling over the surface types, disregarding periods that air masses has spent over mixing layer.

Table S1. Data coverage (%) for daily measurements of ions, metals, SMPS and APS in 2015.

	March	April	May	June	July	August	September	October	March-October
Ions	96.8	100	96.8	100	83.9	83.9	100	61.3	90.2
Metals	96.8	96.7	96.8	100	83.9	83.9	100	61.3	89.8
SMPS	67.7	100	93.5	90	87.1	93.5	80	38.7	81.2
APS	100	100	100	100	96.8	93.5	93.3	67.7	93.9

Table S2. Diagnostics for bootstrap (BS) mapping and displacement (DISP) for the 9-factor solution. Factor 1: secondary, Factor 2: sea salt, Factor 3: trace metals, Factor 4: nucleation, Factor 5: mineral dust_1, Factor 6: anthropogenic, Factor 7: mineral dust_2, Factor 8: biogenic, Factor 9: blowing snow. BS mapped over ~80% of 7 factors and 35% of 2 factors. The two factors are nucleation and blowing snow. It is normal to see the high uncertainties for nucleation and blowing snow factors as they mainly present in the lowest and highest diameter ranges, respectively. From the DISP diagnostics, the uncertainties for DISP can be interpreted and the number of factors is appropriate.

(a) BS Mapping (20 in total):

	Factor 1	Factor 2	Factor 3	Factor 4	Factor 5	Factor 6	Factor 7	Factor 8	Factor 9	Unmapped
Boot Factor 1	20	0	0	0	0	0	0	0	0	0
Boot Factor 2	0	20	0	0	0	0	0	0	0	0
Boot Factor 3	0	0	15	0	1	0	0	0	0	4
Boot Factor 4	1	0	0	7	0	2	0	2	0	8
Boot Factor 5	0	0	0	0	18	2	0	0	0	0
Boot Factor 6	0	0	0	0	0	20	0	0	0	0
Boot Factor 7	0	0	0	0	1	0	19	0	0	0
Boot Factor 8	1	0	0	0	0	0	0	19	0	0
Boot Factor 9	2	0	0	0	1	2	1	0	7	7

(b) DISP Diagnostics:

Error Code:	0								
Largest Decrease in Q:	0								
%dQ:	0								
Swaps by Factor:	0	0	0	0	0	0	0	0	0

Table S3. PMF model performance for ions, metals and PM_{10} using the nine-factor solution.

	Intercept	Slope	SE	R^2	KS Test Stat	KS Test P Value
NH_4^+	0	0.84	0.02	0.76	0.06	0.37
K^+	0	0.69	0.01	0.72	0.06	0.41
Mg^{2+}	0	0.88	0.01	0.94	0.05	0.55
Cl^-	0.01	0.83	0.13	0.9	0.07	0.18
NO_3^-	0.01	0.31	0.01	0.37	0.07	0.17
Oxalate	0	0.24	0	0.62	0.12	0
MSA	0	0.74	0.01	0.78	0.23	0
Br^-	0	0.16	0	0.31	0.11	0.01
ss-Na^+	-0.01	1.02	0.08	0.95	0.1	0.02
ss-Ca^{2+}	0	1.02	0	0.95	0.1	0.02
nss-Na^+	0	0.94	0	0.84	0.22	0
nss-Ca^{2+}	0	0.94	0.01	0.84	0.22	0
ss-SO_4^{2-}	0	1.02	0.02	0.95	0.1	0.02
mineral-SO_4^{2-}	0	0.94	0	0.84	0.22	0
bio-SO_4^{2-}	0	0.74	0.02	0.78	0.23	0
Al	0	0.85	0	0.88	0.08	0.11
Ti	0	0.15	0	0.15	0.04	0.76
V	0	0.25	0	0.24	0.08	0.1
Cr	0	0.17	0	0.18	0.06	0.29
Mn	0	0.65	0	0.73	0.1	0.02
Fe	0.01	0.25	0.01	0.25	0.13	0
Ni	0	0.35	0	0.32	0.23	0
Cu	0	0.15	0	0.12	0.1	0.02
Zn	0	0.4	0	0.25	0.32	0
As	0	0.51	0	0.62	0.16	0
Cd	0	0.15	0	0.28	0.33	0
Ba	0	0.01	0	0.01	0.15	0
La	0	0.7	0	0.82	0.15	0
Ce	0	0.75	0	0.82	0.1	0.01
Pb	0	0.55	0	0.75	0.14	0
PM_{10}	-0.21	0.90	0.92	0.79	0.10	0.02

REFERENCES

(1) Paatero, P.; Tapper, U. Positive Matrix Factorization: A Non-Negative Factor Model with Optimal Utilization of Error Estimates of Data Values. *Environmetrics* **1994**, *5* (2), 111–126. https://doi.org/10.1002/env.3170050203.

(2) Ogulei D; Hopke, P. K.; Wallace LA. Analysis of Indoor Particle Size Distributions in an Occupied Townhouse Using Positive Matrix Factorization. *Indoor Air* **2006**, *16* (3), 204–215. https://doi.org/10.1111/j.1600-0668.2006.00418.x.

(3) Ogulei, D.; Hopke, P. K.; Zhou, L.; Patrick Pancras, J.; Nair, N.; Ondov, J. M. Source Apportionment of Baltimore Aerosol from Combined Size Distribution and Chemical Composition Data. *Atmospheric Environment* **2006**, *40*, 396–410. https://doi.org/10.1016/j.atmosenv.2005.11.075.

(4) Harrison, R. M.; Beddows, D. C. S.; Dall'Osto, M. PMF Analysis of Wide-Range Particle Size Spectra Collected on a Major Highway. *Environmental Science & Technology* **2011**, *45* (13), 5522–5528. https://doi.org/10.1021/es2006622.

(5) Emami, F.; Hopke, P. K. Effect of Adding Variables on Rotational Ambiguity in Positive Matrix Factorization Solutions. *Chemometrics and Intelligent Laboratory Systems* **2017**, *162*, 198–202. https://doi.org/10.1016/j.chemolab.2017.01.012.

(6) Dall´Osto, M.; Beddows, D. C. S.; Tunved, P.; Krejci, R.; Ström, J.; Hansson, H.-C.; Yoon, Y. J.; Park, K.-T.; Becagli, S.; Udisti, R.; Onasch, T.; O´Dowd, C. D.; Simó, R.; Harrison, R. M. Arctic Sea Ice Melt Leads to Atmospheric New Particle Formation. *Scientific Reports* **2017**, *7* (1), 3318. https://doi.org/10.1038/s41598-017-03328-1.

(7) Lee, H.; Lee, K.; Lunder, C. R.; Krejci, R.; Aas, W.; Park, J.; Park, K.-T.; Lee, B. Y.; Yoon, Y. J.; Park, K. Atmospheric New Particle Formation Characteristics in the Arctic as Measured at Mount Zeppelin, Svalbard, from 2016 to 2018. *Atmospheric Chemistry and Physics* **2020**, *20* (21), 13425–13441. https://doi.org/10.5194/acp-20-13425-2020.

(8) Ricard, V.; Jaffrezo, J.-L.; Kerminen, V.-M.; Hillamo, R. E.; Teinilä, K.; Maenhaut, W. Size Distributions and Modal Parameters of Aerosol Constituents in Northern Finland during the European Arctic Aerosol Study. *Journal of Geophysical Research: Atmospheres* **2002**, *107* (D14), AAC 4-1-AAC 4-18. https://doi.org/10.1029/2001JD001130.

(9) Song, C.; Dall'Osto, M.; Lupi, A.; Mazzola, M.; Traversi, R.; Becagli, S.; Gilardoni, S.; Vratolis, S.; Yttri, K. E.; Beddows, D. C. S.; Schmale, J.; Brean, J.; Kramawijaya, A. G.; Harrison, R. M.; Shi, Z. Differentiation of Coarse-Mode Anthropogenic, Marine and Dust Particles in the High Arctic Islands of Svalbard. *Atmospheric Chemistry and Physics* **2021**, *21* (14), 11317–11335. https://doi.org/10.5194/acp-21-11317-2021.

(10) Yu, J. Z.; Huang, X.-F.; Xu, J.; Hu, M. When Aerosol Sulfate Goes Up, So Does Oxalate: Implication for the Formation Mechanisms of Oxalate. *Environmental Science & Technology* **2005**, *39* (1), 128–133. https://doi.org/10.1021/es049559f.

(11) Zhou, Y.; Huang, X. H.; Bian, Q.; Griffith, S. M.; Louie, P. K. K.; Yu, J. Z. Sources and Atmospheric Processes Impacting Oxalate at a Suburban Coastal Site in Hong Kong: Insights Inferred from 1 Year Hourly Measurements. *Journal of Geophysical Research: Atmospheres* **2015**, *120* (18), 9772–9788. https://doi.org/10.1002/2015JD023531.

(12) Moroni, B.; Ritter, C.; Crocchianti, S.; Markowicz, K.; Mazzola, M.; Becagli, S.; Traversi, R.; Krejci, R.; Tunved, P.; Cappelletti, D. Individual Particle Characteristics, Optical Properties and Evolution of an Extreme Long-Range Transported Biomass Burning Event in the

European Arctic (Ny-Ålesund, Svalbard Islands). *Journal of Geophysical Research: Atmospheres* **2020**, *125* (5), e2019JD031535. https://doi.org/10.1029/2019JD031535.
(13) Viana, M.; Amato, F.; Alastuey, A.; Querol, X.; Moreno, T.; García Dos Santos, S.; Herce, M. D.; Fernández-Patier, R. Chemical Tracers of Particulate Emissions from Commercial Shipping. *Environmental Science & Technology* **2009**, *43* (19), 7472–7477. https://doi.org/10.1021/es901558t.
(14) Millero, F. J. The Physical Chemistry of Seawater. *Annual Review of Earth and Planetary Sciences* **1974**, *2* (1), 101–150. https://doi.org/10.1146/annurev.ea.02.050174.000533.
(15) Henderson, P.; Henderson, G.; others. *Cambridge Handbook of Earth Science Data*; Cambridge University Press, 2009.
(16) Becagli, S.; Caiazzo, L.; di Iorio, T.; di Sarra, A.; Meloni, D.; Muscari, G.; Pace, G.; Severi, M.; Traversi, R. New Insights on Metals in the Arctic Aerosol in a Climate Changing World. *Science of The Total Environment* **2020**, *741*, 140511. https://doi.org/10.1016/j.scitotenv.2020.140511.
(17) Abbatt, J. P. D.; Leaitch, W. R.; Aliabadi, A. A.; Bertram, A. K.; Blanchet, J.-P.; Boivin-Rioux, A.; Bozem, H.; Burkart, J.; Chang, R. Y. W.; Charette, J.; Chaubey, J. P.; Christensen, R. J.; Cirisan, A.; Collins, D. B.; Croft, B.; Dionne, J.; Evans, G. J.; Fletcher, C. G.; Gal\'{\i}, M.; Ghahremaninezhad, R.; Girard, E.; Gong, W.; Gosselin, M.; Gourdal, M.; Hanna, S. J.; Hayashida, H.; Herber, A. B.; Hesaraki, S.; Hoor, P.; Huang, L.; Hussherr, R.; Irish, V. E.; Keita, S. A.; Kodros, J. K.; Köllner, F.; Kolonjari, F.; Kunkel, D.; Ladino, L. A.; Law, K.; Levasseur, M.; Libois, Q.; Liggio, J.; Lizotte, M.; Macdonald, K. M.; Mahmood, R.; Martin, R. v; Mason, R. H.; Miller, L. A.; Moravek, A.; Mortenson, E.; Mungall, E. L.; Murphy, J. G.; Namazi, M.; Norman, A.-L.; O'Neill, N. T.; Pierce, J. R.; Russell, L. M.; Schneider, J.; Schulz, H.; Sharma, S.; Si, M.; Staebler, R. M.; Steiner, N. S.; Thomas, J. L.; von Salzen, K.; Wentzell, J. J. B.; Willis, M. D.; Wentworth, G. R.; Xu, J.-W.; Yakobi-Hancock, J. D. Overview Paper: New Insights into Aerosol and Climate in the Arctic. *Atmospheric Chemistry and Physics* **2019**, *19* (4), 2527–2560. https://doi.org/10.5194/acp-19-2527-2019.
(18) Dall'Osto, M.; Beddows, D. C. S.; Tunved, P.; Harrison, R. M.; Lupi, A.; Vitale, V.; Becagli, S.; Traversi, R.; Park, K.-T.; Yoon, Y. J.; Massling, A.; Skov, H.; Lange, R.; Strom, J.; Krejci, R. Simultaneous Measurements of Aerosol Size Distributions at Three Sites in the European High Arctic. *Atmospheric Chemistry and Physics* **2019**, *19* (11), 7377–7395. https://doi.org/10.5194/acp-19-7377-2019.
(19) Myriokefalitakis, S.; Tsigaridis, K.; Mihalopoulos, N.; Sciare, J.; Nenes, A.; Kawamura, K.; Segers, A.; Kanakidou, M. In-Cloud Oxalate Formation in the Global Troposphere: A 3-D Modeling Study. *Atmospheric Chemistry and Physics* **2011**, *11* (12), 5761–5782. https://doi.org/10.5194/acp-11-5761-2011.
(20) Spolaor, A.; Barbaro, E.; Cappelletti, D.; Turetta, C.; Mazzola, M.; Giardi, F.; Björkman, M. P.; Lucchetta, F.; Dallo, F.; Pfaffhuber, K. A.; Angot, H.; Dommergue, A.; Maturilli, M.; Saiz-Lopez, A.; Barbante, C.; Cairns, W. R. L. Diurnal Cycle of Iodine, Bromine, and Mercury Concentrations in Svalbard Surface Snow. *Atmospheric Chemistry and Physics* **2019**, *19* (20), 13325–13339. https://doi.org/10.5194/acp-19-13325-2019.
